# Algorithmic approaches to protein-protein interaction site prediction

**DOI:** 10.1186/s13015-015-0033-9

**Published:** 2015-02-15

**Authors:** Tristan T Aumentado-Armstrong, Bogdan Istrate, Robert A Murgita

**Affiliations:** Department of Anatomy and Cell Biology, McGill University, Montreal, Canada; School of Computer Science, McGill University, Montreal, Canada; Department of Microbiology and Immunology, McGill University, Montreal, Canada

**Keywords:** Prediction algorithm, Protein-protein interaction, Protein-protein interface, Protein-protein binding, Feature selection, Protein structure, Interface types, Machine learning, Biological databases, Homology

## Abstract

Interaction sites on protein surfaces mediate virtually all biological activities, and their identification holds promise for disease treatment and drug design. Novel algorithmic approaches for the prediction of these sites have been produced at a rapid rate, and the field has seen significant advancement over the past decade. However, the most current methods have not yet been reviewed in a systematic and comprehensive fashion. Herein, we describe the intricacies of the biological theory, datasets, and features required for modern protein-protein interaction site (PPIS) prediction, and present an integrative analysis of the state-of-the-art algorithms and their performance. First, the major sources of data used by predictors are reviewed, including training sets, evaluation sets, and methods for their procurement. Then, the features employed and their importance in the biological characterization of PPISs are explored. This is followed by a discussion of the methodologies adopted in contemporary prediction programs, as well as their relative performance on the datasets most recently used for evaluation. In addition, the potential utility that PPIS identification holds for rational drug design, hotspot prediction, and computational molecular docking is described. Finally, an analysis of the most promising areas for future development of the field is presented.

## Background

Interactions between proteins drive the majority of cellular mechanisms, including signal transduction, metabolism, and senescence, among others. The identification of the surface residues mediating these processes, known as protein-protein interaction sites (PPISs), holds great therapeutic potential for the rational design of molecules modulating or mimicking their effects. Further, knowledge of the interacting sites can aid in other domains of computational biology, including PPI network construction and simulated docking. However, biochemical identification methods, such as experimental alanine scanning mutagenesis and crystallographic complex determination, are costly and time-consuming [1,2]. Such methods also consider only sites of the complex under examination, disregarding disparate sites involved in other interactions [3,4]. In response to these shortcomings, computational methods for the prediction of PPISs have been developed, starting with Jones and Thornton’s pioneering analysis of surface patches [5,6], and many predictors have since been published [7-43], utilizing a wide variety of algorithmic approaches to the problem.

This review will first provide a systematic analysis of the features and datasets used in PPIS prediction, from both a theoretical and application-oriented standpoint. An examination of the algorithms used in a selected set of the most recent PPIS predictors is also given, showcasing the diversity of the latest methods employed in this endeavour, as well as the potential for combining or extending them. This is complemented by a comparative evaluation of the performance of these predictors. Because it most accurately simulates the missing information inherent in real-world applications, we focus on the general case of PPIS prediction: using only a single unbound protein structure, without knowledge of an interacting partner, to predict the binding site of that protein at the amino acid scale. Finally, the applications of PPIS prediction and promising areas for future improvements are also discussed.

## Interaction types

Virtually all cellular machinery is composed of proteins, whose functions are mediated through biomolecular interactions; these serve to transmit signals and traffic molecular materials throughout the cell, as well as to form larger multimeric complexes capable of more complex behaviour [44,45]. These interactions occur predominantly at conserved interfaces on the surfaces of the folded protein structures, often resulting in allosteric changes in the flexible conformations of the partners that alter their functions [46]. The potential biomedical utility of interface identification makes the prediction of PPISs a critical endeavour, which necessitates theoretical knowledge of the various types of protein-protein interaction sites.

One major distinction is between obligate complexes, which, by definition, do not form their characteristic structure *in vivo* unless bound, and non-obligate complexes, which can exist as stable monomers [47,48]; complexes are also divided along a continuum between transient and permanent interactions [49], based on temporal length or energetic strength [48,50-52]. Many methods are designed to predict transient interfaces (TIs) [8,17,22,28,35-37,53,54], as they have greater pharmacological relevance, particularly for signal transduction cascades [50,52]. However, TIs tend to be more difficult to predict than permanent interfaces [18,20,23,27,33,39,52,55], possibly resulting from the weaker nature of the interaction manifesting itself as a weaker signal in the properties defining the interacting residues [27,33,52]. However, the existence of fewer training examples due to data gathering difficulties may also play a role [47,49,56-58]. In general, TIs are less evolutionarily conserved than permanent interfaces [50,59-61], but more conserved than the rest of the protein surface [48]. Further, TIs tend to be more compact [51] and richer in water (i.e. more prone to water-mediated binding) [51,62,63]. They also differ in residue propensities [18], including fewer hydrophobic [64] and more polar residues [65]. Thus, unsurprisingly, training on one interface type to predict on the other tends to decrease scores [18,33], though this is sometimes not the case [13]. Generally, analysis of transient versus permanent complexes uses predefined sets [13,60,66] or programs designed to separate them [52,67-69].

All interfaces have distinctive “core” and “rim” regions, with core regions exhibiting lower sequence entropy (higher conservation) than rim [70], as well as reduced tolerance for water and decreased polarity [71-73]. The core of interfaces may be more readily predictable than the rim [7,33], likely for the same reason (i.e. stronger characterizing signal) that permanent PPISs are easier to predict than TIs.

Upon binding, many proteins undergo conformational changes [51,74], which some interface predictors take into account [4,37,38,75]. Large-scale conformational change, such as a disorder-to-order shift [76], is believed to make prediction more difficult for computational protein-protein docking [77,78]; some PPIS predictors also have this difficulty [7,16,25,28,34,35], though several do not [12,32,42].

## Datasets

### Sources of training data

The majority of predictors based on machine learning (ML) rely on sets of structural information to train their learners, mainly curated from the PDB [79]. However, in the process of mining this database, it is necessary to filter out molecules that are not of sufficient quality or utility for use in the training set [37,80]. An overview of these filters is presented in Table 1.

**Table 1:** **Filters used to curate protedatasets for use in training PPIS predictors, including the reasoning behind their use, the methods and specific software used to implement them, as well as references detailing the predictors making use thereof**

**Filter**	**Reason**	**Method**	**Software**	**Used By**
Exclusion of non-biological complexes	Avoid training on complexes not present *in vivo*	Check against other database	PQS [81], PISA [82,83]	[7,12,32,42]
Resolution	Low resolution structures may be inaccurate	PDB filtering	In-House	[7,20,29,37]
Canonical AAs	Most programs cannot handle non-canonical amino acids			[7,12,37,42]
Redundancy	Reduce overfitting	Sequence similarity cutoff	BLAST [84], PISCES [85,86], CD-HIT [87,88]	[7,12,20,29,37,38, 42]
		Removal of members of same superfamily	SCOP [89,90]	[40]
		Similarity clustering with representative structure	In-House	[15,31,32]
Specialized databases	Pre-filtered databases are more reliable	Use of database	ProtInDB [91], Piccolo [92], Negatome [93], iPfam [94,95], 3did [96]	[12,38]
Chain Length	Ensure removal of fragments and peptides	PDB filtering; UniPROT [97] annotation and mapping to PDB [98]	In-House	[7,12,20,29,37,38, 42]
Only X-ray Crystal Structures	NMR are harder to validate, less precise, and more difficult to process [99-101]	PDB filtering	In-House	[37,42]
No antibody-antigen interactions	Ag-Ab complexes bind on different principles than PPIs [9,16,102]			[37,40,103]

### Performance benchmark datasets

Due to the wide range of techniques used by existing predictors, an objective performance evaluation requires the use of standardized datasets that encompass as much of the diversity of proteins and interfaces as possible [75,104,105]. This includes sets such as the Docking Benchmark set, created in 2003 [106] and updated 3 times since its inception [77,107,108], which has seen significant use among PPIS predictors in its original, unedited form [12,18,28,103,109], as well as in modified forms [12,42,42,109]. All widely used modern testing sets are presented in Table 2.

**Table 2:** **Datasets Used to Evaluate Predictors in Table** 4 **, including the source from which they were derived, as well as the publication in which they were created using the requirements in the “Description” column**

**Label**	**Name**	**Derived from**	**Source**	**Description**	**Creator**	**Year**
A	DB3-188	DB 3.0	[77]	$\mathcal {S}_{i} < 40\% $; >50	[31]	2010
B	DS56B	CAPRI	[78]	Targets 1-27 Bound	[31]	2010
C	DS56U	CAPRI	[78]	Targets 1-27 Unbound	[31]	2010
D	NI1	PDB	[79]	$\mathcal {S}_{i} < 70\% $; ≤3.5; 100≤ ≤800;	[37]	2014
				*N*_100_≥1; Excl. *Ag-Ab*; Non-obligate; MC		
E	NI2	PDB	[79]	$\mathcal {S}_{i} < 70\% $; ≤3.5; 100≤ ≤800;	[37]	2014
				*N*_100_≥2; Excl. *Ag-Ab*; Non-obligate; MC		
F	PlaneDimers	Mintz et al.	[110]	Planar PPI; 20*%*<*MSA*_*i*_<90*%*;	[33]	2011
				Excl. MBPs, *Ag-Ab*, VS; >100; Perm		
*	Dimers	Mintz et al.	[110]	Clustered on seq. similarity; Excl.	[33]	2011
				MBPs, *Ag-Ab*, VS; >100; Perm		
G	TransComp_1	DB 4.0	[108]	“Simple” (low conf. change); Non-obligate	[33]	2011
*	TransComp_2	CAPRI	[111]	Not in TransComp_1; Non-obligate	[33]	2011
H	W025	DB 1.0/2.0	[106,107]	Excl. *Ag-Ab*, enzyme interactions	[41]	2006
I	S435	PDB	[79]	PQS filtered; $\mathcal {S}_{i} < 50\% $; >30;	[39]	2007
				Excl. NA, MBPs, VS, NMR		
J	S149	PDB	[79]	PQS filtered; $\mathcal {S}_{i} < 50\% $; >30; Excl.	[39]	2007
				NA, MBPs, VS, NMR; *N*_*H*_(*S*435)		
*	S21a	S149	[39]	Nonredundant; MC	[39]	2007
K	S58	PDB	[79]	$\mathcal {S}_{i} < 30\% $; ≤3.0; >100;	[7]	2012
				Excl. NA, ligands; *N*_*H*_(*S*435)		
L	3DS	3did	[112]	$\mathcal {S}_{i} < 25\% $; >50	[38]	2012
M	B100	DB 3.0	[77]	Excl. *Ag-Ab*	[40]	2011
N	BM180	PDB	[79]	$\mathcal {S}_{i} < 20\% $; ≤3.0; >20; Excl.	[13]	2005
				NMR; Divided into 4 sub-types		
*	S1	PDB	[79]	$\mathcal {S}_{i} < 50\% $; 10< <30; Disordered	[113]	2009
				short; Excl. MBPs, NA; Disprot filtered		
*	S2	PDB	[79]	$\mathcal {S}_{i} < 50\% $; >30; Disordered	[113]	2009
				long; Excl. MBPs, NA; Disprot filtered		
*	DS24Carl	PDB	[79]	>20; 8 Perm + 16 Non-obligate	[66]	2008

The “Label” column defines the alphabetic character used to refer to the dataset in Table 4. “ ∗” in the “Label” column signifies that the set is not presented in Table 4 as it is not widely used. $\mathcal {S}_{i}$ is the sequence identity redundancy cutoff,  is the amino acid length of the chain,  is the resolution cutoff in angstroms, *N*_100_≥*n* requires that the number of interface residues per 100 residues in a given protein to be greater than *n*, *Ag-Ab* refers to antigen-antibody complexes, *M* *S* *A*_*i*_ is the sequence identity redundancy cutoff for chains in an MSA, VS refers to Viral Subunits, NA refers to Nucleic Acids, *N*_*H*_(*x*) refers to a set being non-homologous to set *x*, MC denotes that both the monomer and the complex to which it belongs are known.

## Features

Characterizing features have been used to predict PPISs since the founding of the field [5], and have since been combined with ML algorithms of increasing sophistication. While no single feature appears to possess sufficient information to allow prediction on its own, certain attributes have been consistently favoured, such as conservation and hydrophobicity. Recently, the use of databases of precalculated features, instead of on-the-fly calculations, has gained popularity, via databases such as AAIndex [114,115] and Sting [116]. The most popular features, as well as references detailing their utility and procurement, are presented in Table 3.

**Table 3:** **Compilation of selected software/methods used to compute features described in** Features **section, including the predictors utilizing each and the publications describing their utility and recommending their use**

**Feature**	**Software/method**	**Used by**	**Recommended by**
Accessible Surface Area	PSAIA [117], SurfRace [118], DSSP [119], NACCESS [120], BALL [121], SSpro4 [122], MSMS [123]	Li [29], Sikic [8], Li [38], JET [23], PresCont [33], PredUs [32], Prollo & Meller [39], RAD-T [37]	Chen [16], Jones & Thornton [5], Hoskins [124], Ezkurdia [75]
Conservation	HSSP [125,126], ConSurf-HSSP [66], PSI-BLAST [127], Scorecons [128], Rate4Site [129], AL2CO [130]	Li [29], JET [23], BindML [12], RAD-T [37], PresCont [33], VORFFIP [40]	Zhou & Shan [30], RAD-T [37]
Depth Index	DPX [131] in PSAIA [117]	Sikic [8], Li [38], VORFFIP [40]	Sikic [8]
Protrusion	CX [132] in PSAIA [117]	Sikic [8], Li [38], VORFFIP [40], RAD-T [37], Chen [7]	Jones & Thornton [5]
Hydrophobicity	PSAIA [117], QUITE [133], Fauchère & Pliska [134]	Sikic [8], Ezkurdia [75], RAD-T [37], VORFFIP [40], PresCont [33]	Neuvirth [35], Jones & Thornton [5]
Secondary Structure	DSSP [119], SSpro4 [122]	Sikic [8], VORFFIP [40], Li [38]	Neuvirth [35], Hoskins [124], Ofran & Rost [22]
Propensity	Dong [18], PresCont [33], RAD-T [37]	Dong [18], Jones & Thornton [5], PresCont [33], RAD-T [37]	Conte [64], Zhou & Shan [30], Crowley & Golovin [135], Jones & Thornton [5], Dong [18], Sillerud [136], Levy [137], Tuncbag [58]
Disorder	VSL2 [138], RONN [139]	Li [38], RAD-T [37]	Wright [140], Dunker [141], Liu [142], Iakoucheva [143]
Curvature	Coleman method [144], SurfRace [118]	Li [38], RAD-T [37], PresCont [33]	Jones & Thornton [5]
B-Factors	Curated from PDB [79], Yuan [145]	RAD-T [37], Chung [15], VORFFIP [40], Liu method [54]	Ezkurdia [54,75]
Electrostatic Potential	APBS [146], DelPhi [147], FoldX [148,149]	RAD-T [37], Bradford & Westhead [13], Sting-LDA-WNA [42]	RAD-T [37]
Side-chain Conformational Entropy	FoldX [148,149]	VORFFIP [40]	Cole & Warwicker [150], Liang [28]
Residue Contact Frequencies	PredUs [32]	PredUs [32]	PredUs [32]
Atomic Probability Density Map Features	Yu [151], X-SITE [152]	Chen [7]	Chen [7]
Energy of Solvation	Fernandez-Recio method [105], Fiorucci method [9] using APBS [146]	Fiorucci [9], RAD-T [37]	Fiorucci [9], RAD-T [37]

### Evolutionary metrics

#### Conservation scores

It has been clearly established that measures of evolutionary rate and conservation carry information about ligand-binding and catalytic sites on proteins [153-155]. However, PPIS conservation has been debated, with some claiming that interface residues are not highly differentially conserved compared to the rest of the protein [156,157] or that evolutionary measures alone are of limited predictive accuracy [22,39,158,159]. Some PPIS predictors do not use conservation [7,8] or note that its use makes little difference [27,39]. On the other hand, a number of analytical studies have found greater conservation among interface residues [16,30,55,160] and that conservation holds predictive utility [40,161]. Such conflicting conclusions may be a result of the different datasets and methods used to compute conservation.

Regardless, numerous PPIS predictors have made heavy use of residue conservation features [13,16,21,22,33,35-38,40,41,162], including several based almost solely on evolutionary metrics [12,20,23,52,163], suggesting such measures do indeed have significant predictive power. In terms of generality, evolutionary methods can be more widely applicable than physicochemical ones, as the latter characteristics may differ markedly between functional sites, whereas conservation patterns in the former may be more easily recognizable [164]. Further, sequence conservation-based predictors do not require structural information [14,22,36]. However, since evolutionary methods are non-specific enough to be used for identification of any type of functional site [165-169], this lack of specialization may reduce performance for PPIS prediction specifically [4]. Unsurprisingly, conservation is not helpful for interfaces selected by non-evolutionary means, such as antigen-antibody complexes [16,102] (which require separate specialized predictors [170]).

Predictors employ a number of methods to extract evolutionary features. Commonly, a multiple sequence alignment (MSA) from BLAST [84] or PSI-BLAST [127] is used to derive conservation scores, in conjunction with substitution matrices (e.g. Dayhoff [171], BLOSUM [172], or position-specific) [13,14,33,38,41], often in combination with information theoretic measures (generally based on Shannon entropy [173]) [18,27,33,39]. Some predictors [14,37,40] use previously developed programs for computing conservation [128-130,174-176].

#### Sequence homology

Predictors that are more reliant on evolutionary metrics tend to have more complex methods of deriving evolutionary conservation scores [12,20,23,52]. JET [23] builds off the evolutionary trace approach [177-180], constructing phylogenetic distance trees to extract a residue ranking based on evolutionary importance, then using this to derive likely PPISs. Similarly, BindML [12] uses local MSAs, specialized substitution matrices, and phylogenetic tree construction to obtain the likelihood that a given patch MSA belongs to a PPIS (described below). HomPPI [20] derives linear combinations of BLAST statistics correlated to conservation score in known interfaces, allowing an estimation of conservation in unknown interfaces.

#### Structural homology

While the above methods are largely dependent on sequence-based measures of evolutionary information, structural conservation has also been shown to be useful for distinguishing protein binding sites [31,160,181,182] and even general functional sites [183]. Importantly, structure evolves more slowly than sequence and may have more powerful signals for conservation [184,185]. Predictors using structural homology have verified this proposition [15,21,31,32,66,162,186].

The main issue with the use of structural homologs, or with predictors requiring structural information in general, is the paucity of usable structures [15,47,105,187], particularly when considering the relatively small size of the PDB (∼80,000 structures, including redundancy) compared to the number of sequences known (∼17 million non-redundant sequences) [36,188], though this can be partly circumvented by using local (rather than global) structural homologies [34]. However, studies on the properties of the interfaces themselves have found that their structural space is degenerate [189], that templates for the majority of known interactions exist [185,189], and that interfaces are conserved across structure space [31]. This bodes well for structure-based PPIS prediction, as it suggests that numerically limited interface examples can cover most potential queries, despite incomplete structural [187,188,190] and interaction type (or quaternary fold) [3,191] coverage. The potential contribution of homology models [190,192-194] and growing structural coverage [188] further justify using structure.

### Physicochemical characteristics

Physicochemical properties have long been used for interface prediction [102]. The observed trend of increased hydrophobicity in interfaces compared to non-interacting surface patches [5,195-197], as well as the proposed pattern of a highly hydrophobic central region surrounded by polar residues [71,198], has been used with success in several recent predictors [8,33,37,40,75]. Additionally, electrostatic potential [13,42] and energy of desolvation [9] have shown utility as discriminative properties of PPISs [13,37,42]. B-factors (Debye-Waller temperature factors) [15,40] and disorder measures [37,38] in prediction software are also useful, with interface sites shown to be more disordered [38,140,141] yet also appearing to have lower B-factors than other surface patches [15,28,35,54,150]. Further, the decreased flexibility implied by decreasing B-factors is confirmed by the observation that interface residues minimize the entropic cost of complex formation [28] by avoiding the sampling of alternative side-chain rotamers [40,150].

### Statistical measures

#### Propensity

Residue propensity, which measures PPIS amino acid composition, has been used in the characterization of interface types (e.g. homodimers versus heterodimers [16,199], permanent versus transient [18,80], biological versus non-biological [200]) or subtypes (e.g. core versus rim [72,201]), in hotspot prediction [160,202], and evolution [203], as well as in PPIS prediction [13,28,33,35,37,41,204,205]. In general, polar amino acids are statistically disfavoured in interfaces sites, with the exception of arginine [18,35]. Further, while propensity differences are relatively minor between complex types, greater favouring of cysteine and leucine has been observed in permanent (but not transient) interfaces [18].

In general, predictors [13,35,37] use a variant of the following equation to compute propensity based on amino acid frequencies:

$$ prop(r) = \frac{{count}_{int}(r)/|Interface|}{{count}_{sur}(r)/|Surface|} $$

where *count*_*int*_(*r*) and *count*_*sur*_(*r*) are the frequencies of occurrence of residue *r* in interfaces and on protein surfaces, respectively, and |*Interface*| and |*Surface*| denote the sizes of these sets. More sophisticated extensions include using combinations of residues [33] (combinatorially expanding the number of possible *r* values, though amino acid categories can reduce this [206]), weighting by accessibility [28,205], or computing binary profiles [18].

#### Atomic contact probability density maps

The packing preferences and geometries of atomic protein structures have long been studied as a characterizing feature of association [207], via probability density maps (PDMs) describing likelihoods of contacts [7,151,152]. Advantageously, PDMs can be derived from the intra-molecular contacts in the protein interior and are hence less limited by the structural information available [7,152]. While contacts in the protein interior differ from those in interfaces (e.g. artefactual interactions from structural constraints [152] or greater contribution of electrostatics to folding than binding [208]), these differences appear to be relatively minor [196,209-211], particularly when the interface core is mainly considered [137].

Recently, 3D PDMs were used as input features for PPIS prediction [7]. By projecting onto a previously described coordinate system [152], interacting contacts can then be added to the density map, allowing preservation of both magnitude and direction. Based on a similar method applied to protein folding [212], “co-incidental" interactions (due to proximal atoms forced together by structure constraints) [7,151] were filtered out.

These density maps can then serve as PPIS prediction features: given query protein *P*, feature vector *v*_*i*_= <*a*_*i*1_,…,*a*_*in*_> ∀ *i*∈*P*, where *a*_*ij*_ represents the distance-weighted normalized sum of PDM values of atom *i* with interacting atom type *j*, and *n* is the number of interacting atom types (31, based on current works [7,151]). The resulting attribute vector set *V*={*v*_*i*_ ∀ *i*∈*P*} is amenable to ML-based PPIS prediction.

### Structural geometry

#### Solvent accessible surface area

Often chosen as one of the most discriminatory features by a wide array of predictors [8,23,29,31-33,37-39], the Solvent Accessible Surface Area (SASA) of residues is thought to be of significant importance in PPIS prediction. Jones and Thornton [5,6] were among the first to suggest that high solvent accessibility is indicative of a residue’s participation in an interaction site. The relationship was further validated by Chen and Zhou on a set of 1256 nonhomologous protein chains [16], as well as by Hoskins et al. [124], who also accounted for the relative contributions of both the main and side chains (polar and non-polar) of the protein.

#### 3-D characteristics

PPISs have long been thought to posses distinct 3-dimensional characteristics that allow them to be distinguished from the rest of the protein surface [5]. In particular, curvature has been singled out as an important 3D structural characteristic [33,37,38,186], with interface sites thought to be significantly more concave than the rest of the protein surface, lending stability and specificity to the interface [38].

This has, however, been disputed in the literature [11], with the prevailing opinion appearing to favour shape complementarity, in which one of the proteins in a complex contains a concave binding site, while the interaction site on its partner exhibits convexity, in order to bind “snugly” [213]. Interestingly, Nooren and Thornton [48] showed that transient PPISs not only tend to be more planar than their permanent counterparts, but that there is a gradient even within transient sites, with “stronger” sites exhibiting greater curvature than those in “weaker” transient interactions.

Similarly, secondary structural characteristics have been used in several predictors [8,22,38,102], but have also elicited dispute regarding their utility and biological interpretation. Specifically, some studies [35,124] have found that *β*-sheets are favoured in interface sites while *α*-helices are more prevalent over the rest of the protein surface, though others disagree [38,102].

#### Depth and protrusion indices

As discussed previously, interfaces are rich in hydrophobic residues, which is superficially incongruous with the finding that interfaces tend to have a higher solvent accessibility than non-interface patches. This has been explained by Li et al. [38], who, along with Sikic et al. [8] and Segura et al. [40], found the depth and protrusion features to be the most highly discriminatory features of their respective predictors. Thus, interface residues tend to have a higher average depth index (are more deeply buried), while maintaining a higher side-chain protrusion (leading to the observed increase in solvent accessible surface area) [38].

## Algorithmic approaches

While there exist previous reviews on PPIS prediction [4,75,109,214,215], these did not have access to the most recent algorithmic advances. As such, we provide a systematic overview of these techniques, in the hope that future predictors can employ these methodologies as a foundation for the creation of more sophisticated predictors.

### Feature selection

Feature selection is an indispensable part of ML, in which redundant and irrelevant attributes are removed from the feature set to ensure predictor efficacy [216]. Redundancy provides no new information (but potentially creates noise), is computationally inefficient, and overweights the contribution of that information, leading to overfitting and thus lower prediction scores.

#### Genetic-race search

The computational power required to check all possible combinations of features renders such an approach impractical. To efficiently search this space, a method of feature selection termed Genetic-Race Search (GRS) [37] was recently used, combining a genetic algorithm [217] with RACE search [218].

Each feature set (“individual”) is represented as a bitstring, and the fitness of the individual is defined as its Matthews Correlation Coefficient (MCC) [219] after leave-one-out-cross-validation (LCV). A population of these individuals is then iteratively altered by three operations: mutation (with preference for less fit individuals), selection, and crossover (preferentially choosing higher scoring individuals) [217].

At every iteration, the top *k* individuals (“elites”) are saved and used to augment evaluation efficiency via Hoeffding races [220]. As LCV is performed for a given individual, its empirical mean $\bar {x}$ is continuously updated as each protein is analyzed. The *k*th elite (i.e. the least fit elite) and its fitness score *f*_*EK*_ is continually compared to $\bar {x}$ as LCV goes on. If (1−*δ*)*%* certainty that *f*_*EK*_>*μ* is reached, evaluation of the current individual can be halted, as it is statistically incapable of entering the elites.

To compute this certainty, the two-tailed symmetric distribution derived from Hoeffding’s original bounds [221] can be used [220]:

$$ P(|\bar{x}-\mu| > \epsilon) < 2 \exp\left({\frac{-2n \epsilon^{2}}{B^{2} }}\right) $$

where the random variable (MCC) is bounded with range *B* and *n* is the number of samples. Clearly, (1−*δ*)% certainty that the maximum distance between $\bar {x}$ and *μ* is less than *ε* requires $P(|\bar {x}-\mu | > \epsilon) < \delta $. Thus:

$$ \epsilon = \sqrt{\frac{B^{2}}{2n}\ln\left(\frac{2}{\delta}\right)} $$

Hence, given *n* measurements, *μ* is within *ε* of $\bar {x}$ with (1−*δ*)% certainty. Notably, Hoeffding bounds do not rely on a particular underlying probability distribution and are thus widely applicable to different fitness measures with high conservatism (though reducing *δ* can mitigate this).

#### MRMR-IFS

The minimum redundancy maximal relevance (MRMR) method [222,223] ranks features by importance via mutual information [224], which measures the non-linear dependence between random variables. The ranked variables are then combined with incremental feature selection (IFS), which chooses an attribute set by stepwise construction of unique feature subsets [225,226]. For example, in the recent PPIS prediction method by Li et al. [38], MRMR-IFS was used to reduce a set of over 700 features to only 51.

First, the mutual information *I*(*X*,*Y*) between two features *X* and *Y*, which serves as a measure of non-linear correlation, is defined as follows:

$$ I(X,Y)= \iint\limits_{X\,Y} P(x,y) \log_{b}\left(\frac{P(x,y)}{P(x)P(y)}\right)dy\,dx $$

where *P*(*x*,*y*) is the joint and *P*(*x*) and *P*(*y*) are the marginal probability density functions.

To calculate the relevance *D* of a feature *f* to the class *c* being predicted, one can compute *D*=*I*(*f*,*c*). Then, given a feature set *Ω*, the redundancy *R* of *f* to the values in *Ω* is defined as the average information held by *f* that is already in *Ω*:

$$ R = \frac{1}{|\Omega|} \sum\limits_{f_{i} \in \Omega} I\left(f,f_{i}\right) $$

MRMR then orders the feature set *Ω* by sequential addition to a set of already selected attributes, *Ω*_*s*_, from the “remainder” set *Ω*_*t*_=*Ω*∖*Ω*_*s*_. At every step, the best feature *f*_*b*_ to add to *Ω*_*s*_ is the one that best balances high *D* with low *R*:

$$ f_{b} = \underset{f_{j}\in \Omega_{t}}{\arg\max} \left[ I(f_{j},c) - \frac{1}{|\Omega_{s}|} \sum\limits_{f_{i}\in\Omega_{s}} I\left(f_{j},f_{i}\right) \right] $$

The order of placement into *Ω*_*s*_ is the output ranking of MRMR.

To apply IFS to the MRMR ranking, Li et al. [38] then built subsets of features by iteratively adding attributes in the order of the MRMR ranking and chose the feature subset with the maximal MCC score via ten-fold cross-validation.

#### Principal component analysis

Alternatively, principal component analysis (PCA), a method of information-preserving dimensionality reduction, can be used [227,228]. The number of principal component (PC) vectors required to account for any amount of the original variance in the data can be calculated (via the PCA eigenvalues), allowing control of the trade-off between high dimensionality and relevance to the predicted class. Further, the PCs are orthogonal and hence linearly uncorrelated, greatly reducing feature redundancy. The features of this new space (with the PCs as basis vectors) can then be used as input to a machine learner.

There are a variety of criteria for selecting the appropriate number of eigenvectors to use, including the widely used method of accounting for an arbitrarily chosen amount of variance sufficient to cover the majority of information required for the prediction task [229]. The PPIS predictor by de Moraes et al. [42], for example, chooses the number of PCs necessary to account for 95% of the variance of the data, after removing variables with excessively high linear correlation to each other.

### Surface-interface size relation

The percentage of interacting surface residues is not constant with respect to protein size; rather, it has long been known that it follows a non-linear distribution (e.g. exponential regression line) [6,230,231]. However, this information has only seldom been applied to PPIS prediction [16,23], or even treated as a linearly changing proportion [13,35], despite results showing that this “size bias” carries significant predictive power on its own [103]. One potential application to ML-based predictors is dynamically setting the prediction threshold such that the proportion of predicted active residues matches the estimated prior distribution for the query protein. Such intelligent biasing of the learner might even be extended by looking for patterns in the interface-surface residue ratio in different types of proteins as well.

### Homology-based predictors

#### PredUs

The PredUs algorithm is based on the observation that PPISs are conserved across structural space [31,32], allowing known sites of structural homologs to be mapped onto query proteins. The initial version of PredUs [31] used an interfacial score *ς* with an empirically derived threshold. First, for a given query protein *Q*, a set of structural neighbours *N*_*i*_ was derived using Ska [232,233], where each *N*_*i*_ is in complex with a partner *P*_*i*_. By bringing *P*_*i*_ into the coordinate space of *Q* via superposition for every *N*_*i*_, the sum of contacts in the new space gives a frequency *f*_*r*_ for every residue *r*∈*Q*. The interfacial score for each residue is then given by:

$$\varsigma(r) = \frac{1}{1+\exp\left(\frac{-f_{r} + f_{max}/2}{f_{max}/10} \right) } $$

where *f*_*max*_ is the maximum value across the whole structure.

The second version of PredUs added an ML layer to their homology-based method [32]. For a given *Q*, a map of contact frequencies was computed for each surface residue as shown above. Then, for every *r*∈*Q*, a surface patch consisting of *r* and its closest spatial neighbours was constructed. For each patch, a feature vector of contact frequencies and SASA values (per amino acid) was derived, and a support vector machine (SVM) [234] was used to segregate interacting and non-interacting residues.

#### PrISE

The PrISE (Predictor of Interface Residues using Structural Elements) algorithm addresses several limitations of using whole-protein structural similarity, including the coarseness of global homology measures and the requirement for sufficient numbers of structural neighbours, via local structural conservation information [34].

First, a set *S*(*Q*) of “structural elements” (SEs) is extracted from a query protein *Q*. Every SE *q*_*r*_∈*S*(*Q*) contains the surface residue *r* as its central residue and all of *r*’s surface neighbours, and is represented by an atomic composition frequency histogram of its constituents. A database containing more than 34 million SEs [91] was created to isolate sets of SEs homologous to any *Q*, denoted *H*_*q*_, from a wide variety of proteins based on comparison of their atomic frequency histograms.

For the global predictor, weights *w*(*p*,*q*) were calculated for every pair of elements from *p*∈*H*_*q*_ and *q*∈*S*(*Q*) based on the similarity of the entire protein *Q* to the protein from which *p* was extracted, denoted as *π*(*p*). To evaluate this similarity, the contribution, *cont*(*P*,*R*), was defined as the number of SEs in *R* present in *P*. Thus, the weight for the global predictor is defined as

$$w_{G}(p,q) = cont\left(\pi(p),Z_{Q}\right) $$

where *Z*_*Q*_ is the set union of *H*_*q*_ ∀*q*∈*S*(*Q*).

This was coupled with a local predictor, where the weight was computed as

$$w_{L}(p,q) = cont\left(\pi(p),N_{q}\right) $$

where *N*_*q*_ is the set of SEs with local structural homology to the SE in question, *q*. This is computed by examining every residue *r*_*i*_ in *q*, which has its own associated SE, $S_{r_{i}}$. Next, the set of SEs most similar to $S_{r_{i}}$ is considered, denoted by $R_{r_{i}}$, from the repository of all SEs. Each $\phantom {\dot {i}\!}s_{i} \in R_{r_{i}}$ is locally homologous to some part of *q*, since *r*_*i*_ is a residue in *q* and every SE $\phantom {\dot {i}\!}s_{i} \in R_{r_{i}}$ is homologous to the SE around *r*_*i*_ (i.e. $S_{r_{i}}$). Thus, *N*_*q*_ is defined as:

$$ N_{q} = \bigcup\limits_{r_{i} \in q}R_{r_{i}} $$

The final predictor, called PrISE _*C*_, combined local and global information via a combined weight *w*_*C*_(*p*,*q*)=*w*_*G*_(*p*,*q*)×*w*_*L*_(*p*,*q*). Known PPIS participation of central SE residues was used to compute weights *W*_*C*+_ and *W*_*C*−_ by summing across all *w*_*C*_(*p*,*q*) in which *p* is known to be in an interface (*p*+) and known to not be in an interface (*p*−), respectively. Thus,

$$W_{C+q} = \sum_{p+ \in H_{q}}w_{C}(p,q) \;\;\; \& \;\;\; W_{C-q} = \sum_{p- \in H_{q}}w_{C}(p,q) $$

The probability of a residue interacting is derived from the weights via:

$$P_{C+(q)} = \frac{W_{C+q}}{W_{C+q} + W_{C-q}} $$

#### HomPPI

The foundation of the HomPPI predictor family is the evolutionary conservation of interface residues, derived solely from sequence information. The two HomPPI predictors [20], NPS-HomPPI (Non-Partner Specific) and PS-HomPPI (Partner Specific), depend on the correlation between conservation of interfaces and several BLAST alignment statistics of sequence pairs, discovered by PCA analysis. The conservation is calculated as the correlation coefficient of a prediction made by assigning all interacting residues of a protein in the pair to the corresponding residues on its sequence homolog. The BLAST statistics log(EVal), Positive score and log(LAL) were found to be highly correlated with conservation in Non-Partner Specific interfaces, where EVal is the expectation score, LAL is the Local Alignment Length, and the Positive score is the number of positive matches in the alignment.

The information obtained by PCA was used to create a linear scoring function via Interface Conservation (IC) score, with the NPS predictor using

$$\begin{aligned} {IC}_{NPS}=\beta_{0} &+ \beta_{1} \text{log}(EV\,al) \\ &+ \beta_{2} PosS + \beta_{3} \text{log}(LAL) \end{aligned} $$

where all *β*_*i*_s were chosen to correlate best with the correlation coefficient above.

This *IC*_*NPS*_ score was used to rank homologs of the query protein by their predicted conservation, of which the top ten were chosen to undergo a form of majority vote, where each residue was given a score based on the ratio of positive to negative votes. A threshold for this score was used to determine the interacting residues on the query.

#### BindML

The Binding site prediction by Maximum Likelihood (BindML) approach is based on sequence-derived evolutionary information, though it does use an input structure to choose patches of the query protein to target [12,52]. The first step involves the construction of two amino acid substitution matrices: one describing PPISs or protein binding interfaces (PBIs), and the other describing non-protein binding interfaces (NPBIs) or non-PPISs (NPPISs), via MSAs from iPFAM [94]. These matrices, *M*_*PBI*_ and *M*_*NPBI*_ respectively, are computed by counting substitutions with pairwise alignment sets [235], followed by construction with the BLOSUM method [172].

Then, for a given query protein *Q* with surface residues *S*_*i*_, a set of patches ({*P*_*i*_}) is produced for every *S*_*i*_, based on an empirically chosen radial distance cutoff. For every *P*_*i*_, the corresponding residues (and the aligned columns within the family MSA in which *Q* belongs) are concatenated. This patch MSA is then used to produce two phylogenetic trees for each *P*_*i*_, *T*_*PBI*_(*i*) and *T*_*NPBI*_(*i*), using *M*_*PBI*_ and *M*_*NPBI*_, respectively. This is done with the BIONJ method [236], an extension of the neighbor-joining algorithm [237] that takes the variance of the evolutionary distances into account. A modified version of the PHYML algorithm [238] is then used to compute the log likelihood of the patch and the tree (built with *M*_*PBI*_ or *M*_*NPBI*_) as follows:

$$ \begin{aligned} L_{PBI}(i) &= \log\left(\text{P}(P_{i},T_{PBI}(i)|M_{PBI})\right)\\ L_{NPBI}(i) &= \log\left(\text{P}(P_{i},T_{NPBI}(i)|M_{NPBI})\right) \end{aligned}  $$

A difference score, *dL*(*i*)=*L*_*NPBI*_(*i*)−*L*_*PBI*_(*i*), can then be computed for every surface residue, combined and recast into Z-scores, and finally thresholded to determine whether a given residue is interacting or not.

### Machine learning-based techniques

#### Li et al. 2012 method

The Li et al method [38] uses machine learning on feature vectors derived from sequence windows. These data vectors include amino acid properties (from AAIndex [114*,*^115^]), conservation data, solvent accessibility values, and structural information. First, for every residue in each protein, a peptide is extracted with the residue in question serving as its center, accounting for the local environment of each amino acid. The peptides are labeled as active or inactive based on the label of their central residue and filtered for homology. Then, large feature vectors are extracted to represent each peptide, by extracting and concatenating a variety of attributes per residue, in the order of the peptide. Their dimensionality *D* is given by *D*=*NL*, where *N* is the number of residue-level features and *L* is the length of each peptide (here, 34 and 21, respectively [38]). To remove irrelevant and redundant features from this high dimensional feature space, the MRMR-IFS feature selection procedure is applied (see Feature Selection). In the reduced space, the attribute vectors of the peptides are then used to construct a Random Forest (RF) classifier [239], an ensemble learner based on combining the output of multiple decision trees.

#### PresCont

The PresCont algorithm [33] combines local residue features with environmental information as input to an SVM. The creators of PresCont note their belief that interface prediction will not benefit from the use of a large number of noisy features, and thus make use of just a few important attributes, namely SASA, hydrophobicity, propensity and conservation. Additionally, the weighted average of each of these features over the neighbouring residues using a Euclidean distance cutoff is used. The features are scaled to the range [0,1] and used as input to an SVM with the radial basis kernel function, which constructs a hyperplane capable of optimally separating PPIS and NPPIS feature vectors.

To test the utility of accounting for core-rim differences, the authors used the Intervor [240] algorithm (which computes distance to the interface rim via Voronoi shelling order) with PresCont, detecting differences in propensities (as found previously [198]), but ultimately not recommending use of core residues alone for training when the full PPIS is desired.

#### RAD-T

The Residues on Alternating Decision Trees (RAD-T) algorithm combines supervised ML with representative characteristics from all major feature types [37]. Training data was produced from monomers mapped back from their complexes, but separately crystallized as monomeric structures. This was done to train the learner on proteins in their monomeric conformation, rather than their complexed one, as PPIS predictors will tend to be run on monomeric proteins of interest, produced by crystallization or by modelling, for which the partners or complexes are not known.

The class imbalance problem, caused by the numerical disparity between PPIS and NPPIS training examples, was addressed by resampling the high number of non-interacting examples to match the number of interacting ones in a 1:1 ratio, based on empirically testing different ratios of positive-to-negative results over a variety of machine learners. The resulting set of feature vectors can then be used to optimize a given machine learner by GRS, which will find the feature subset optimally discriminative of PPIS participation for a given learner and dataset.

For ML, RAD-T then uses an alternating decision tree (ADTree) [241*,*^242^], an extension of the classical decision tree that integrates across multiple paths in the tree and makes use of boosting, in which multiple weak learners are used to build a single strong one.

#### VORFFIP

The limited information contained in single residues has often been supplemented with environmental or neighbourhood information [8*,*^38^*,*^39^]. The VORFFIP (Voronoi Random Forest Feedback Interface Predictor) algorithm [40] makes use of atom-resolution 3D Voronoi diagrams [243] to identify spatially neighbouring surface residues, for which features are assigned using structural, energetic, evolutionary, and experimental data. The use of Voronoi diagrams may be better than other approaches as it is based on an implicitly defined “visibility” between residues (avoiding choice of falloff rates and threshold values), and it allows weighting environmental contributions with greater resolution [40].

In addition, the method discriminates and removes outliers, as residues with high probability of PPIS participation are generally in contiguous patches and not surrounded by low probability residues. This is accomplished with the use of a 2-step RF ensemble classifier, each instantiation of which consists of several hundred discrete decision trees, with the first RF utilizing structure-, energy-, and evolutionary-based features for each surface residue, as well as environmental information, and the second RF making use of the scores from the previous step, along with further environmental score-derived metrics.

Both RFs include a weighting function that accounts for the strength of contact *c*_*ij*_ between amino acids *a*_*i*_ and *a*_*j*_ based on their atoms’ positions in the Voronoi diagram, giving greater influence to neighbouring residues with more atomic contacts. This weight is defined as *c*_*ij*_=*N*_*ij*_/*N*_*i*_, where *N*_*ij*_ is the number of contacts (shared facets in the Voronoi diagram) between the atoms of *a*_*i*_ and *a*_*j*_, and *N*_*i*_ is the sum of all atomic contacts made by *a*_*i*_. Once the first RF calculates the probability of residues being involved in interaction sites, several further metrics are created from these predictions. These include the environmental score *es*_*i*_, which weights the scores from the first RF for each neighbouring residue, and Contact Score Vector *csv* features, which account for the contributions of each residue type:

$${es}_{i} = \sum\limits_{j=1}^{n}c_{ij}s_{j} \;\;\;\;\; \& \;\;\;\;\; {csv}_{l} = \sum\limits_{a_{j} \equiv {type}_{l}}c_{ij}s_{j} $$

where *s*_*j*_ is the score of the neighbour residue, *l* is one of the 20 residue types, and residue *a*_*j*_ has type *l*. Once calculated, these scores are added to those from the first-step RF for each residue as input to the second-step RF, generating a revised prediction with reduced outliers, better environmental accounting, and improved prediction performance.

#### Sting-LDA-WNA

The method by de Moraes et al., designated Sting-LDA-WNA, combines PCA-based recombination of neighbourhood-averaged feature vectors with amino acid-specific linear classifiers [42]. The Sting database [116] was used to extract a residue-level feature vector for each amino acid in every protein. To consider the local environment of each residue, weighted neighbour averages were utilized via the two approaches of Porollo and Meller [39]:

$$V^{S}_{\text{WNA}} = \sum\limits_{i=0}^{N} V_{i} \text{RSA}_{i} \;\;\;\;\;\&\;\;\;\;\; V^{d}_{\text{WNA}} = V_{0} + \sum\limits_{i=1}^{N} \frac{V_{i}}{d_{i}} $$

where *N* is the number of neighbouring residues (within a sphere of 15Å, based on previous work [39*,*^244^]), *V*_*i*_ is the feature value of the *i*th neighbour, *d*_*i*_ is its distance from the target residue, and RSA_*i*_ is its relative solvent accessibility. Finally, feature selection was performed by (1) removal of attributes with high linear correlation and (2) PCA, with sufficient principal components to permit 95% of the variance to be explained (see Feature Selection), resulting in a feature space with low dimensionality and redundancy.

Linear discriminant analysis (LDA) was then used to derive a hyperplane capable of separating input vectors based on the class labels of the training data [245*,*^246^]. Importantly, the authors used amino-acid specific classifiers (i.e. a different LDA classifier was built and then applied for each of the canonical amino acid types), leading to higher predictive ability.

#### Chen et al. 2012 method

Unlike most other predictors, the method by Chen et al. operates on the atomic level, utilizing PDMs (encoding the probabilistic “strength” of interaction of a given protein atom with every other potential atom type) to produce atom-level feature vectors [7]. To account for neighbourhood information, Chen et al. also add the distance-weighted atomic density values for the neighbouring atoms, followed by normalization. Along with a measure of the unoccupied Van der Waals volume around a given atom, this gives a set of per-atom feature vectors for any protein.

These vectors were then used for ML via a feed-forward artificial neural network (ANN) with a sigmoid transfer function [247*,*^248^] and the resilient back-propagation algorithm [249]. Separate ANN classifiers for each protein atom type were trained and their outputs combined, with training designed to maximize MCC. Further, the ensemble-based bootstrap aggregation (bagging) algorithm was employed to counter the class imbalance [250].

The final ensemble of atom-specific, bagging ANNs could then predict the interface atoms of a given query protein surface. To turn these into residue-level predictions, high-confidence atomic predictions were treated as “seeds”, and any surrounding atoms of even moderate confidence were assigned to be part of an atomic interacting patch. Any residues with a high proportion of its atoms being part of such a patch were considered interacting.

### Ensemble predictors

Given the disparate approaches and information sources applied to PPIS prediction, it is natural that combining multiple methods should increase scores. For example, using amino acid-specific [42] and atom-specific [7] classifier ensembles permits the reduction of noise and better separation between residues/atoms that likely have different properties differentiating them in interface sites. Similarly, the combination of local and global structural homology-based predictors in PrISE [34] and the two-step RF of VORFFIP [40] both increase scores over the independent counterparts. Some predictors also find increased scores upon combination with previous methods, as in WHISCYMATE [41] and Combined BindML [12].

More integrative approaches utilize meta-predictors, which combine multiple predictors into a single output score. Meta-PPISP [25] combines cons-PPISP [16], Promate [35], and PINUP [28] via a linear regression model taking local environment into account.

By adding SPPIDER [39] and PPI-PRED [13] to that list, metaPPI [24] uses the frequency with which its constituent predictors consider a residue interacting to predict continuous PPIS patches, by iteratively adding surface vertices. More recently, CPORT [17] uses a consensus approach to combine several predictors, by adding residues if they pass a predictor-specific threshold for any given predictor. In all cases tested so far, these meta-predictors see an increase in score and robustness compared to the individual predictors.

## Evaluation

A comparative evaluation of PPIS predictors has been performed in previous reviews [109*,*^214^]; as such, we focus on information regarding only the most recent predictors, shown in Table 4.

**Table 4:** **Comparative evaluation of recent predictors**

**PredName**	**Year**	**Label**	**Dataset**	**Precision**	**Recall**	**Accuracy**	**AUC**	**MCC**	**F1**	**Source**
ProMate	2004	A	DB3-188 ^*†*^	36.5	30.3	77.1	67.7	19.5	33.1	[34]
		B	DS56B	31.9	27.3	76.7	63.3	15.6	29.4	
		C	DS56U	28.7	27.3	76.6	62.7	14.0	28.0	
		E	NI2	40.0	93.9	∗	∗	13.6	56.1	[37]
		F	PlaneDimers	∗	∗	∗	68.0	18.0	∗	[33]
		G	TransComp_1	∗	∗	∗	70.0	20.0	∗	
ConsPPISP	2005	A	DB3-188 ^*†*^	46.5	30.6	80.4	73.2	26.7	36.9	[34]
		B	DS56B	39.8	36.1	78.9	72.6	25.2	37.9	
		C	DS56U	37.4	34.5	79.5	71.2	23.8	35.9	
		E	NI2	49.3	32.2	∗	∗	14.7	39.0	[37]
PINUP	2006	A	DB3-188 ^*†*^	40.7	34.7	78.3	66.0	24.6	37.5	[34]
		B	DS56B	37.3	31.9	78.4	63.7	21.7	34.4	
		C	DS56U	30.4	30.1	76.9	60.0	16.4	30.2	
		E	NI2	52.9	28.5	∗	∗	15.1	37.0	[37]
WHISCY	2006	H	W025	39.0	27.0	∗	∗	27.0	∗	[40]
metaPPISP	2007	A	DB3-188 ^*†*^	49.0	26.7	81.1	74.6	26.2	34.6	[34]
		B	DS56B	43.3	25.8	80.8	74.4	22.9	32.3	
		C	DS56U	38.9	24.0	81.1	71.5	20.2	29.7	
		E	NI2	54.7	25.5	∗	∗	16.6	34.8	[37]
		F	PlaneDimers	∗	∗	∗	54.0	4.0	∗	[33]
		G	TransComp_1	∗	∗	∗	78.0	31.0	∗	
PIER	2007	E	NI2	44.1	83.6	∗	∗	23.0	57.7	[37]
SPPIDER	2007	J	S149	63.7	60.3	∗	76.0	42.0	∗	[40]
		F	PlaneDimers	∗	∗	∗	80.0	33.0	∗	[33]
		G	TransComp_1	∗	∗	∗	68.0	15.0	∗	
Sikic	2009	L	3DS	63.4	78.3	65.3	∗	30.8	∗	[38]
PredUs	2010	A	DB3-188	43.6	45.7	∗	∗	∗	∗	[31]
		B	DS56B	41.5	42.2	∗	∗	∗	∗	
		C	DS56U	39.8	44.6	∗	∗	∗	∗	
	2011	A	DB3-188	50.3	57.5	72.6	73.9	34.5	53.0	[32]
		B	DS56B	43.0	53.0	72.1	71.3	29.0	47.4	
		C	DS56U	43.3	53.6	73.2	72.9	30.4	47.9	
		K	S58	45.5	57.6	78.5	∗	37.7	50.8	[7]
VORFFIP	2011	J	S149	63.4	74.7	∗	90.0	58.0	∗	[40]
		H	W025	42.0	47.0	∗	∗	38.0	∗	
		M	B100	45.0	56.0	∗	∗	42.0	49.0	
HomPPI	2011	N	BM180 ^1^	∗	58.0	85.0	∗	44.0	∗	[20]
			BM180 ^2^	∗	48.0	84.0	∗	42.0	∗	
			BM180 ^3^	∗	71.0	86.0	∗	60.0	∗	
			BM180 ^4^	∗	73.0	91.0	∗	65.0	∗	
PrISE	2012	A	DB3-188 ^*†*^	48.0	43.2	80.6	77.2	33.8	45.5	[34]
		B	DS56B	46.1	45.4	80.9	77.6	34.1	45.7	
		C	DS56U	43.7	44.0	81.2	75.5	32.6	43.8	
Li mRMR-IFS	2012	L	3DS	65.3	79.0	67.3	∗	34.8	∗	[38]
Chen PDM-ML	2012	I	S435 ^*‡*^	51.2	66.2	75.9	∗	42.0	57.8	[7]
		J	S149 ^*§*^	51.9	67.7	75.3	∗	42.3	58.8	
		K	S58	44.6	65.4	77.7	∗	40.3	53.0	
PresCont	2012	F	PlaneDimers	∗	∗	∗	80.0	33.0	∗	[33]
		G	TransComp_1	∗	∗	∗	69.0	17.0	∗	
RAD-T	2014	A	DB3-188	28.5	64.7	65.2	∗	22.2	35.5	[37]
		D	NI1	33.8	80.5	51.8	∗	20.1	46.4	
		E	NI2	44.7	80.9	59.1	∗	26.4	57.6	

^*†*^refers to the DB3-188 set excluding 2VIS; ^*‡*^refers to the S435 set excluding 3 proteins due to obsolescence or absence; ^*§*^refers to the S149 set excluding 7 proteins due to existence in training set. BM180 ^1^ are transient enzyme-inhibitor complexes, BM180 ^2^ are transient non-enzyme- inhibitor complexes, BM180 ^3^ are obligate hetero-dimers and BM180 ^1^ are obligate homo-dimers.

Objective evaluation of PPIS predictor performance is made difficult by the varying definitions of interaction sites and accessible surface residues in the literature, the lack of available servers for all predictors, the adoption of varying training and testing datasets, and the different metrics used for evaluation [4*,*^47^]. We partially circumvent these problems by considering the performance of each predictor across a variety of test sets based on literature values.

### Assessment measures

PPIS predictors are generally judged with a number of standard performance metrics, including sensitivity (recall, true positive rate, or coverage), precision, and specificity:

$$\begin{aligned} & Accuracy &&= \frac{T_{P}+T_{N}}{T_{P}+F_{P}+T_{N}+F_{N}} \\ & Precision &&= \frac{T_{P}}{T_{P}+F_{P}} \\ & Sensitivity &&= \frac{T_{P}}{T_{P}+F_{N}}\\ & Specificity &&= \frac{T_{N}}{T_{N}+F_{P}} \end{aligned} $$

where *T*_*P*_, *T*_*N*_, *F*_*P*_, and *F*_*N*_ denote true and false positives and negatives, respectively.

Measures designed to balance between false negative and positive rates include *F*_1_ and MCC [219]:

$$\begin{aligned} F_{1} &= 2\frac{precision \cdot recall}{precision + recall} \\ \\ MCC &= \frac{T_{P}\cdot T_{N} - F_{P}\cdot F_{N}}{\alpha} \end{aligned} $$

where:

$${\alpha=\sqrt{(T_{P}+F_{P})(T_{P}+F_{N})(T_{N}+F_{P})(T_{N}+F_{N})}} $$

Similarly, the receiver operator curve (ROC), which is a plot of *sensitivity* versus 1−*specificity* derived by varying the classifier prediction threshold, can be used to compute the area under the ROC curve (AUROC/AUC) [251], which is especially useful for identifying artificially “inflated” performance (e.g. higher sensitivity at the expense of specificity) and for being decision threshold independent [252].

In general, similar to the lack of consensus in interface definitions and datasets, there is no standard criteria for performance assessment [47]. Given that some false positive predictions may be correct (due to the paucity of crystallized complexes), patch-specific performance metrics (i.e. assessing the correct answer in a local patch around an interface in question, such as by the Sørensen-Dice index [253*,*^254^]) may be used, though this poorly accounts for false positives. While other evaluation methods have been devised [16*,*^35^], computing the statistics above per residue and averaging across the dataset appears to be the most objective and easily comparable method.

The authors note that even the more balanced measures should not be solely relied on (e.g. MCC may favour overprediction in PPIS prediction [214] and underprediction elsewhere [255]) and that predictor performance should be viewed holistically across as many metrics as possible, as balancing performance metrics is domain-dependent [47*,*^255^]. When considering PPIS prediction for mimetic drug design, slight underprediction may be desirable, as it will likely find the better discriminated core residues [7*,*^33^], from which the remaining PPIS can be inferred (rather than “guessing” which of many allegedly “active” residues is even interacting).

### Comparative evaluation

While it is difficult to draw conclusions from the differing performance of the predictors, we can nevertheless observe some trends that may be explained by the biological theory discussed previously. For example, while transient datasets (such as TransComp_1) generally garner lower scores than permanent ones (such as PlaneDimers), this is not perfectly followed (Table 4), possibly due to the difficulty in defining a threshold on the transient-permanent continuum. Some sets (e.g. S149) may be intrinsically more predictable, as evidenced by higher scores across all predictors; others achieve better results only on certain types of predictors (e.g. DB3-188 on structural homology-based predictors). To achieve high scores on specialized testing datasets, predictors often require either specializations of their own, or inherent characteristics that permit accurate classification (e.g. ANCHOR’s [10] specialization for disordered proteins and HomPPI’s [20] lack of requirement for structural information allow them both to successfully predict on the S1/2 disordered sets). Theoretically, unbound structures are more difficult to predict on than bound monomers (due to the conformational disparity between the two sets); this is largely confirmed by differing results on the DS56B/U sets, as well as generally lower scores on unbound sets (Table 4). Overall, we find that there has been significant progress in the predictive abilities of the predictors over the last decade across diverse interaction types and datasets.

## Future directions

Though the field of PPIS prediction has been steadily improving in accuracy and sophistication over time, challenges remain before scores sufficient to permit its many potential applications can be achieved.

### Accounting for interface type and subclass

The classification of interfaces between transient, permanent, and obligate, as well as within interfaces as core and rim structures, has been extensively studied from a theoretical standpoint. However, with some exceptions [20*,*^33^*,*^52^], this information has been algorithmically underutilized for PPIS prediction. Focusing on datasets of a particular complex type, combining interface classification with interaction likelihood prediction, and integrating learning of the different properties of the interface core and rim into PPIS prediction are just a few examples of promising areas that are currently under investigation.

### Closer examination of datasets

As with much biological data, protein structural datasets are non-standardized and virtually all structure-based predictors have varying criteria for processing and filtering this data. The types of proteins that are the least predictable, or the difference between large but heterogeneous training sets versus smaller but cleaner ones, have not been comprehensively examined. Specialized training sets per query protein, whether by structural, sequence-level, or functional data, are worth exploring as well. Potentially, unsupervised learning could be applied to extract hidden patterns within the data, possibly contributing to further analysis of the relation between interaction types, protein categories, and the feature space. Further, methods accounting for systemic biases in the PDB (e.g. under-representation of membrane/disordered proteins) may also improve robustness [256*,*^257^].

### Improved benchmarking

More comprehensive and standardized benchmarking, as noted in other reviews [75], is essential to advancing the field. Additionally, to facilitate comparative evaluation of performance, as well as to ensure that improvements are not statistical anomalies, the authors suggest that significance testing be applied to future published work.

### Utilizing ensemble approaches

The numerous methods developed for PPIS prediction often utilize diverse sources of information and varied techniques, suggesting that methods of combining approaches are promising. Current meta-predictors tend to use relatively straightforward methods of combining their constituents [17*,*^24^*,*^25^]; ensemble techniques that could take spatial relations into account, such as graph-based or random field models, could be applied for this purpose [258*,*^259^]. The strengths, weaknesses, and specializations of the various approaches should also be taken into account. For instance, homology-based predictors (e.g. PredUS [32] or HomPPI [20]), which can predict exceedingly well when homologues are available but fail otherwise, could be combined with a more general, machine-learning based predictor to complement it in cases where homology information is missing. On a smaller scale, ensemble methods utilizing a set of predictors (e.g. residue-specific [42], atom-specific [7], bagging-based [7]) appear to be useful for reducing noise and making better use of the available information contained within the protein.

### Integrating other areas of computational biology

Greater integration of PPIS prediction with other areas of computational biology also holds promise. The relatively small number of crystallized structures suggests PPIS predictions based on structural characteristics may not be helpful when such information is not present; however, the use of molecular modeling could prove useful in mitigating this problem. Other areas of bioinformatics are also being applied to assist PPIS prediction, such as molecular docking [11*,*^19^*,*^53^*,*^260^].

### Further application of computational techniques

Several areas of PPIS prediction could benefit from the use of more sophisticated computational learning techniques. For feature selection and extraction, current methods can be combined (e.g. MRMR or PCA with GRS) or extended (e.g. empirical Bernstein bounds [261] with GRS, non-linear component analysis [262], or autoencoders [263]). The class imbalance problem has been recently circumvented via bagging [7], but semi-supervised learning could also be applied, as seen for hotspot [264*,*^265^] and pairwise protein interaction prediction [266], with minimal changes to the features used. Indeed, as “non-interacting” residues may be mislabelled (since every possible protein complex is certainly not known), semi-supervised learning methods for handling this problem are even more applicable [267*,*^268^].

Recently, methods for accounting for neighbourhood information have become more prevalent, including averaging across the local environment [7*,*^33^], Voronoi diagrams [40], and feature concatenation [8*,*^38^], each providing gains in predictive ability. This suggests that methods for multi-scale machine learning could prove effective [258*,*^259^]. Machine learning techniques for multi-class classification [269*,*^270^] (e.g. separating core vs. rim vs. surface) also hold potential for improvement.

## Applications

The identification of interacting residues on protein surfaces holds potential for use in diverse fields across biology and medicine. One of the most related problems is the elucidation of the residues inside PPISs that account for the major change in free binding energy upon complex formation, known as hotspots (HSs) [271*,*^272^]. ML-based PPIS predictors have already been successfully used for HS prediction by simply altering the training set [161]. Interface predictions may be used to narrow the search space of HS predictors, as HSs tend to localize to the interface core [33*,*^273^]. For the same reason, putative HS residues could be used to “seed” interface site predictions. Similarly, knowledge of a PPIS can be used to guide mutagenesis experiments to more promising sites, reducing the expense and time required for a whole-protein analysis.

Most importantly, knowledge of PPISs and HSs can be used for rational design of therapeutics and biomolecules by serving as a template for the *de novo* creation of small molecules with enhanced efficacy and selectivity. Mimetics of the interaction sites of well-known molecules have been successfully built [274*,*^275^], the process of which could be significantly expedited by the knowledge of putative interaction sites, as it would allow rational construction of a mimetic compound without requiring mass screening. The design of novel interface sites has also shown promise for the construction of new functional biomolecules [276*,*^277^].

Another interesting application is in assisting computational protein-protein docking. Docking without prior knowledge of the interaction sites of the proteins in question (i.e. *ab initio* docking) has been shown to be more difficult due to the staggering search space dimensionality involved [278]. Information-driven docking [279] can mitigate this problem by utilizing mass spectroscopy and interaction data, the latter of which is often difficult to obtain, but can be provided by PPIS prediction. This approach has already proven successful in both high-resolution docking [17*,*^24^*,*^41^] and coarse mass docking experiments [280]. Additionally, PPIS-driven coarse docking studies could assist in large-scale PPI network creation [281], as well as alignment of such networks for functional ortholog identification [282*-*284].

## Conclusion

The field of protein-protein interaction site prediction has grown significantly since the pioneering work of Jones and Thornton, and is now poised to bring great benefit to other problems in biomedical science, particularly rational drug design. This growth has, however, brought several issues to the forefront, including the need for standardized testing sets and evaluation metrics to ensure that objective comparisons of performance can be carried out. The field has seen numerous algorithmic advances over the past few years, building on decades of theoretical biology, and we expect that combining and extending these algorithms will be a considerable source of improvement in the future. As such, we have undertaken an exhaustive analysis of the state-of-the-art algorithms presently in use and their performance, as well as an exploration of the datasets and features employed by current predictors. We believe that future advances will bring predictors capable of significantly contributing to biomedical and pharmaceutical science.

## References

[1] Krüger DM, Gohlke H (2010). Drugscoreppi webserver: fast and accurate in silico alanine scanning for scoring protein–protein interactions. Nucleic Acids Res..

[2] Bradshaw RT, Patel BH, Tate EW, Leatherbarrow RJ, Gould IR (2011). Comparing experimental and computational alanine scanning techniques for probing a prototypical protein–protein interaction. Protein Eng Des Sel..

[3] Aloy P, Russell RB (2004). Ten thousand interactions for the molecular biologist. Nat Biotechnol..

[4] Porollo A, Meller J (2012). Computational methods for prediction of protein-protein interaction sites.. Protein-Protein Interactions-Computational and Experimental Tools; W. Cai and H. Hong, Eds. InTech..

[5] Jones S, Thornton JM (1997). Analysis of protein-protein interaction sites using surface patches. J Mol Biol..

[6] Jones S, Thornton JM (1997). Prediction of protein-protein interaction sites using patch analysis. J Mol Biol..

[7] Chen C-T, Peng H-P, Jian J-W, Tsai K-C, Chang J-Y, Yang E-W (2012). Protein-protein interaction site predictions with three-dimensional probability distributions of interacting atoms on protein surfaces. PloS one..

[8] Šikić M, Tomić S, Vlahoviček K (2009). Prediction of protein–protein interaction sites in sequences and 3d structures by random forests. PLoS Comput Biol..

[9] Fiorucci S, Zacharias M (2010). Prediction of protein-protein interaction sites using electrostatic desolvation profiles. Biophys J..

[10] Dosztányi Z, Mészáros B, Simon I (2009). Anchor: web server for predicting protein binding regions in disordered proteins. Bioinformatics..

[11] Martin J, Lavery R (2012). Arbitrary protein- protein docking targets biologically relevant interfaces. BMC Biophys..

[12] La D, Kihara D (2012). A novel method for protein–protein interaction site prediction using phylogenetic substitution models. Proteins: Struct Funct Bioinform..

[13] Bradford JR, Westhead DR (2005). Improved prediction of protein–protein binding sites using a support vector machines approach. Bioinformatics..

[14] Chen X-W, Jeong JC (2009). Sequence-based prediction of protein interaction sites with an integrative method. Bioinformatics..

[15] Chung J-L, Wang W, Bourne PE (2006). Exploiting sequence and structure homologs to identify protein–protein binding sites. Proteins: Struct Funct Bioinform..

[16] Chen H, Zhou H-X (2005). Prediction of interface residues in protein–protein complexes by a consensus neural network method: test against nmr data. Proteins: Struct Funct Bioinform..

[17] de Vries SJ, Bonvin AM (2011). Cport: a consensus interface predictor and its performance in prediction-driven docking with haddock. PLoS One..

[18] Dong Q, Wang X, Lin L, Guan Y (2007). Exploiting residue-level and profile-level interface propensities for usage in binding sites prediction of proteins. BMC Bioinform..

[19] Fernández-Recio J, Totrov M, Abagyan R (2004). Identification of protein–protein interaction sites from docking energy landscapes. J Mol Biol..

[20] Xue LC, Dobbs D, Honavar V (2011). Homppi: a class of sequence homology based protein-protein interface prediction methods. BMC Bioinform..

[21] Shoemaker BA, Zhang D, Thangudu RR, Tyagi M, Fong JH, Marchler-Bauer A (2009). Inferred biomolecular interaction server—a web server to analyze and predict protein interacting partners and binding sites. Nucleic Acids Res..

[22] Ofran Y, Rost B (2007). Isis: interaction sites identified from sequence. Bioinformatics..

[23] Engelen S, Trojan LA, Sacquin-Mora S, Lavery R, Carbone A (2009). Joint evolutionary trees: a large-scale method to predict protein interfaces based on sequence sampling. PLoS Comput Biol..

[24] Huang B, Schroeder M (2008). Using protein binding site prediction to improve protein docking. Gene..

[25] Qin S, Zhou H-X (2007). meta-ppisp: a meta web server for protein-protein interaction site prediction. Bioinformatics..

[26] Tjong H, Qin S, Zhou H-X (2007). Pi2pe: protein interface/interior prediction engine. Nucleic Acids Res..

[27] Kufareva I, Budagyan L, Raush E, Totrov M, Abagyan R (2007). Pier: protein interface recognition for structural proteomics. Proteins: Struct Funct Bioinform..

[28] Liang S, Zhang C, Liu S, Zhou Y (2006). Protein binding site prediction using an empirical scoring function. Nucleic Acids Res..

[29] Li M-H, Lin L, Wang X-L, Liu T (2007). Protein–protein interaction site prediction based on conditional random fields. Bioinformatics..

[30] Zhou H-X, Shan Y (2001). Prediction of protein interaction sites from sequence profile and residue neighbor list. Proteins: Struct Funct Bioinform..

[31] Zhang QC, Petrey D, Norel R, Honig BH (2010). Protein interface conservation across structure space. Proc Nat Acad Sci..

[32] Zhang QC, Deng L, Fisher M, Guan J, Honig B, Petrey D. Predus: a web server for predicting protein interfaces using structural neighbors. Nucleic Acids Res. suppl 2; 39:283–7. 10.1093/nar/gkr311 PMC3125747 21609948

[33] Zellner H, Staudigel M, Trenner T, Bittkowski M, Wolowski V (2012). Prescont: Predicting protein-protein interfaces utilizing four residue properties. Proteins: Struct Funct Bioinform..

[34] Jordan RA, Yasser E-M, Dobbs D, Honavar V (2012). Predicting protein-protein interface residues using local surface structural similarity. BMC Bioinformatics..

[35] Neuvirth H, Raz R, Schreiber G (2004). Promate: a structure based prediction program to identify the location of protein–protein binding sites. J Mol Biol..

[36] Murakami Y, Mizuguchi K (2010). Applying the naïve bayes classifier with kernel density estimation to the prediction of protein–protein interaction sites. Bioinformatics..

[37] Bendell CJ, Liu S, Aumentado-Armstrong T, Istrate B, Cernek PT, Khan S (2014). Transient protein-protein interface prediction: datasets, features, algorithms, and the rad-t predictor. BMC Bioinformatics..

[38] Li B-Q, Feng K-Y, Chen L, Huang T, Cai Y-D (2012). Prediction of protein-protein interaction sites by random forest algorithm with mrmr and ifs. PloS one..

[39] Porollo A, Meller J (2007). Prediction-based fingerprints of protein–protein interactions. PROTEINS: Structure Function Bioinform..

[40] Segura J, Jones PF, Fernandez-Fuentes N (2011). Improving the prediction of protein binding sites by combining heterogeneous data and voronoi diagrams. BMC Bioinformatics..

[41] de Vries SJ, van Dijk AD, Bonvin AM (2006). Whiscy: What information does surface conservation yield? application to data-driven docking. Proteins: Struct Funct Bioinform..

[42] de Moraes FR, Neshich IA, Mazoni I, Yano IH, Pereira JG, Salim JA (2014). Improving predictions of protein-protein interfaces by combining amino acid-specific classifiers based on structural and physicochemical descriptors with their weighted neighbor averages. PloS One..

[43] Qiu Z, Wang X (2012). Prediction of protein–protein interaction sites using patch-based residue characterization. J Theor Biol..

[44] Janin J. Basic principles of protein–protein interaction. Computational protein–protein interactions, 2009:1–20.

[45] Alberts B, Johnson A, Raff M, Roberts K, Walter P. Molecular biology of the cell. 2008.

[46] Keskin O, Gursoy A, Ma B, Nussinov R (2008). Principles of protein-protein interactions: what are the preferred ways for proteins to interact?. Chem Rev..

[47] Nussinov R, Schreiber G (2010). Computational Protein-protein Interactions.

[48] Nooren I, Thornton JM (2003). Diversity of protein–protein interactions. EMBO J..

[49] Ozbabacan SEA, Engin HB, Gursoy A, Keskin O (2011). Transient protein–protein interactions. Protein Eng Des Sel..

[50] Amoutzias G, de Peer Y. Single-gene and whole-genome duplications and the evolution of protein-protein interaction networks. Evol Genomics Syst Biol. 2010:413–29.

[51] Perkins JR, Diboun I, Dessailly BH, Lees JG, Orengo C (2010). Transient protein-protein interactions: structural, functional, and network properties. Structure..

[52] La D, Kong M, Hoffman W, Choi YI, Kihara D (2013). Predicting permanent and transient protein–protein interfaces. Proteins: Struct Funct Bioinform..

[53] Fernandez-Recio J, Totrov M, Skorodumov C, Abagyan R (2005). Optimal docking area: a new method for predicting protein–protein interaction sites. PROTEINS: Struct Funct Bioinform..

[54] Liu R, Jiang W, Zhou Y (2010). Identifying protein–protein interaction sites in transient complexes with temperature factor, sequence profile and accessible surface area. Amino Acids..

[55] Choi YS, Yang J-S, Choi Y, Ryu SH, Kim S (2009). Evolutionary conservation in multiple faces of protein interaction. Proteins: Struct Funct Bioinform..

[56] Phizicky EM, Fields S (1995). Protein-protein interactions: methods for detection and analysis. Microbiol Rev..

[57] Chichili V, Kumar V, Sivaraman J (2013). A method to trap transient and weak interacting protein complexes for structural studies. Intrinsically Disordered..

[58] Tuncbag N, Gursoy A, Keskin O (2011). Prediction of protein–protein interactions: unifying evolution and structure at protein interfaces. Phys Biol..

[59] Sprinzak E, Altuvia Y, Margalit H (2006). Characterization and prediction of protein–protein interactions within and between complexes. Proc Nat Acad Sci..

[60] Mintseris J, Weng Z (2005). Structure, function, and evolution of transient and obligate protein–protein interactions. Proc Nat Acad Sci USA..

[61] Brown KR, Jurisica I (2007). Unequal evolutionary conservation of human protein interactions in interologous networks. Genome Biol..

[62] Mihalek I, Reš I, Lichtarge O (2007). On itinerant water molecules and detectability of protein–protein interfaces through comparative analysis of homologues. J Mol Biol..

[63] Levy Y, Onuchic JN (2006). Water mediation in protein folding and molecular recognition. Annu Rev Biophys Biomol Struct..

[64] Conte LL, Chothia C, Janin J (1999). The atomic structure of protein-protein recognition sites. J Mol Biol..

[65] Nooren I, Thornton JM (2003). Structural characterisation and functional significance of transient protein–protein interactions. J Mol Biol..

[66] Carl N, Konc J, Janezic D (2008). Protein surface conservation in binding sites. J Chem Inform Model..

[67] Zhu H, Domingues FS, Sommer I, Lengauer T (2006). Noxclass: prediction of protein-protein interaction types. BMC Bioinformatics..

[68] Aziz M, Maleki M, Rueda L, Raza M, Banerjee S (2011). Prediction of biological protein–protein interactions using atom-type and amino acid properties. Proteomics..

[69] Maleki M, Vasudev G, Rueda L (2013). The role of electrostatic energy in prediction of obligate protein-protein interactions. Proteome Sci..

[70] Guharoy M, Chakrabarti P (2005). Conservation and relative importance of residues across protein-protein interfaces. Proc Nat Acad Sci USA..

[71] Larsen TA, Olson AJ, Goodsell DS (1998). Morphology of protein–protein interfaces. Structure..

[72] Chakrabarti P, Janin J (2002). Dissecting protein–protein recognition sites. Proteins: Struct Funct Bioinform..

[73] Karanicolas J, Corn JE, Chen I, Joachimiak LA, Dym O, Peck SH (2011). A de novo protein binding pair by computational design and directed evolution. Molecular cell..

[74] Truong K, Ikura M (2001). The use of fret imaging microscopy to detect protein–protein interactions and protein conformational changes in vivo. Current Opin Struct Biol..

[75] Ezkurdia I, Bartoli L, Fariselli P, Casadio R, Valencia A, Tress ML (2009). Progress and challenges in predicting protein–protein interaction sites. Brief Bioinformatics..

[76] Janin J, Bahadur RP, Chakrabarti P (2008). Protein–protein interaction and quaternary structure. Q Rev Biophys..

[77] Hwang H, Pierce B, Mintseris J, Janin J, Weng Z (2008). Protein–protein docking benchmark version 3.0. Proteins: Struct Funct Bioinform..

[78] Janin J, Wodak S (2007). The third capri assessment meeting toronto, canada, april 20–21, 2007. Structure..

[79] Bernstein FC, Koetzle TF, Williams GJ, Meyer EF Jr, Brice MD, Rodgers JR (1978). The protein data bank: a computer-based archival file for macromolecular structures. Arch Biochem Biophys..

[80] Ofran Y, Rost B (2003). Analysing six types of protein–protein interfaces. J Mol Biol..

[81] Henrick K, Thornton JM (1998). Pqs: a protein quaternary structure file server. Trends Biochem Sci..

[82] Krissinel E, Henrick K (2007). Inference of macromolecular assemblies from crystalline state. J Mol Biol..

[83] Krissinel E (2010). Crystal contacts as nature’s docking solutions. J Comput Chem..

[84] Altschul SF, Gish W, Miller W, Myers EW, Lipman DJ (1990). Basic local alignment search tool. J Mol Biol..

[85] Wang G, Dunbrack RL (2003). Pisces: a protein sequence culling server. Bioinformatics..

[86] Wang G, Dunbrack RL (2005). Pisces: recent improvements to a pdb sequence culling server. Nucleic Acids Res..

[87] Li W, Godzik A (2006). Cd-hit: a fast program for clustering and comparing large sets of protein or nucleotide sequences. Bioinformatics..

[88] Huang Y, Niu B, Gao Y, Fu L, Li W (2010). Cd-hit suite: a web server for clustering and comparing biological sequences. Bioinformatics..

[89] Murzin AG, Brenner SE, Hubbard T, Chothia C (1995). Scop: a structural classification of proteins database for the investigation of sequences and structures. J Mol Biol..

[90] Andreeva A, Howorth D, Brenner SE, Hubbard TJ, Chothia C, Murzin AG (2004). Scop database in 2004: refinements integrate structure and sequence family data. Nucleic Acids Res..

[91] Jordan R, Wu F, Dobbs D, Honavar V. Protindb: A database of protein-protein interface residues. Iowa State University (In Preparation)

[92] Bickerton GR, Higueruelo AP, Blundell TL (2011). Comprehensive, atomic-level characterization of structurally characterized protein-protein interactions: the piccolo database. BMC Bioinformatics..

[93] Smialowski P, Pagel P, Wong P, Brauner B, Dunger I, Fobo G (2010). The negatome database: a reference set of non-interacting protein pairs. Nucleic Acids Res..

[94] Finn RD, Marshall M, Bateman A (2005). ipfam: visualization of protein–protein interactions in pdb at domain and amino acid resolutions. Bioinformatics..

[95] Finn RD, Miller BL, Clements J, Bateman A (2014). ipfam: a database of protein family and domain interactions found in the protein data bank. Nucleic Acids Res..

[96] Stein A, Russell RB, Aloy P (2005). 3did: interacting protein domains of known three-dimensional structure. Nucleic Acids Res..

[97] Consortium U (2013). Update on activities at the universal protein resource (uniprot) in 2013. Nucleic Acids Res..

[98] Martin AC (2005). Mapping pdb chains to uniprotkb entries. Bioinformatics..

[99] Schneider M, Fu X, Keating AE (2009). X-ray vs. nmr structures as templates for computational protein design. Proteins: Struct Funct Bioinform..

[100] Fan H, Mark AE (2003). Relative stability of protein structures determined by x-ray crystallography or nmr spectroscopy: A molecular dynamics simulation study. PROTEINS: Struct Funct Bioinform..

[101] Lee MR, Kollman PA (2001). Free-energy calculations highlight differences in accuracy between x-ray and nmr structures and add value to protein structure prediction. Structure..

[102] Jones S, Thornton JM (1996). Principles of protein-protein interactions. Proc Nat Acad Sci..

[103] Martin J (2014). Benchmarking protein–protein interface predictions: Why you should care about protein size. Proteins: Struct Funct Bioinform..

[104] Tuncbag N, Kar G, Keskin O, Gursoy A, Nussinov R (2009). A survey of available tools and web servers for analysis of protein–protein interactions and interfaces. Briefings Bioinform..

[105] Fernández-Recio J (2011). Prediction of protein binding sites and hot spots. Wiley Interdiscip Rev Comput Mol Sci..

[106] Chen R, Mintseris J, Janin J, Weng Z (2003). A protein–protein docking benchmark. Proteins: Struct Funct Bioinform..

[107] Mintseris J, Wiehe K, Pierce B, Anderson R, Chen R, Janin J (2005). Protein–protein docking benchmark 2.0: an update. Proteins: Struct Funct Bioinform..

[108] Hwang H, Vreven T, Janin J, Weng Z (2010). Protein–protein docking benchmark version 4.0. Proteins: Struct Funct Bioinform..

[109] Zhou H-X, Qin S (2007). Interaction-site prediction for protein complexes: a critical assessment. Bioinformatics..

[110] Mintz S, Shulman-Peleg A, Wolfson HJ, Nussinov R (2005). Generation and analysis of a protein–protein interface data set with similar chemical and spatial patterns of interactions. Proteins: Struct Funct Bioinform..

[111] Lensink MF, Wodak SJ (2010). Blind predictions of protein interfaces by docking calculations in capri. Proteins: Struct Funct Bioinform..

[112] Stein A, Céol A, Aloy P (2011). 3did: identification and classification of domain-based interactions of known three-dimensional structure. Nucleic Acids Res..

[113] Mészáros B, Simon I, Dosztányi Z (2009). Prediction of protein binding regions in disordered proteins. PLoS Comput Biol..

[114] Kawashima S, Kanehisa M (2000). Aaindex: amino acid index database. Nucleic Acids Res..

[115] Kawashima S, Pokarowski P, Pokarowska M, Kolinski A, Katayama T, Kanehisa M (2008). Aaindex: amino acid index database, progress report 2008. Nucleic Acids Res..

[116] Neshich G, Mazoni I, Oliveira S, Yamagishi M, Kuser-Falcão P, Borro L (2005). The star sting server: a multiplatform environment for protein structure analysis. Genet Mol Res GMR..

[117] Mihel J, Šikić M, Tomić S, Jeren B, Vlahoviček K (2008). Psaia–protein structure and interaction analyzer. BMC Struct Biol..

[118] Tsodikov OV, Record MT, Sergeev YV (2002). Novel computer program for fast exact calculation of accessible and molecular surface areas and average surface curvature. J Comput Chem..

[119] Kabsch W, Sander C (1983). Dictionary of protein secondary structure: pattern recognition of hydrogen-bonded and geometrical features. Biopolymers..

[120] Hubbard SJ, Thornton JM. Naccess. Computer Program, Department of Biochemistry and Molecular Biology, University College London. 1993;2(1).

[121] Hildebrandt A, Dehof AK, Rurainski A, Bertsch A, Schumann M, Toussaint NC (2010). Ball-biochemical algorithms library 1.3. BMC Bioinformatics..

[122] Cheng J, Randall AZ, Sweredoski MJ, Baldi P (2005). Scratch: a protein structure and structural feature prediction server. Nucleic Acids Res..

[123] Sanner MF, Olson AJ, Spehner J-C (1996). Reduced surface: an efficient way to compute molecular surfaces. Biopolymers..

[124] Hoskins J, Lovell S, Blundell TL (2006). An algorithm for predicting protein–protein interaction sites: abnormally exposed amino acid residues and secondary structure elements. Protein Sci..

[125] Sander C, Schneider R (1991). Database of homology-derived protein structures and the structural meaning of sequence alignment. Proteins: Struct Funct Bioinform..

[126] Dodge C, Schneider R, Sander C (1998). The hssp database of protein structure—sequence alignments and family profiles. Nucleic Acids Res..

[127] Altschul SF, Madden TL, Schäffer AA, Zhang J, Zhang Z, Miller W (1997). Gapped blast and psi-blast: a new generation of protein database search programs. Nucleic Acids Res..

[128] Valdar WS (2002). Scoring residue conservation. Proteins: Struct Funct Bioinform..

[129] Pupko T, Bell RE, Mayrose I, Glaser F, Ben-Tal N (2002). Rate4site: an algorithmic tool for the identification of functional regions in proteins by surface mapping of evolutionary determinants within their homologues. Bioinformatics..

[130] Pei J, Grishin NV (2001). Al2co: calculation of positional conservation in a protein sequence alignment. Bioinformatics..

[131] Pintar A, Carugo O, Pongor S (2003). Dpx: for the analysis of the protein core. Bioinformatics..

[132] Pintar A, Carugo O, Pongor S (2002). Cx, an algorithm that identifies protruding atoms in proteins. Bioinformatics..

[133] Lijnzaad P, Berendsen HJ, Argos P (1996). A method for detecting hydrophobic patches on protein surfaces. Proteins: Struct Funct Bioinform..

[134] Fauchere J, Pliska V (1983). Hydrophobic parameters-pi of amino-acid side-chains from the partitioning of n-acetyl-amino-acid amides. Eur J Med Chem..

[135] Crowley PB, Golovin A (2005). Cation– *π* interactions in protein–protein interfaces. Proteins: Struct Funct Bioinform..

[136] Sillerud LO, Larson RS (2005). Design and structure of peptide and peptidomimetic antagonists of protein-protein interaction. Current Protein Peptide Sci..

[137] Levy ED (2010). A simple definition of structural regions in proteins and its use in analyzing interface evolution. J Mol Biol..

[138] Peng K, Radivojac P, Vucetic S, Dunker AK, Obradovic Z (2006). Length-dependent prediction of protein intrinsic disorder. BMC Bioinformatics..

[139] Yang ZR, Thomson R, McNeil P, Esnouf RM (2005). Ronn: the bio-basis function neural network technique applied to the detection of natively disordered regions in proteins. Bioinformatics..

[140] Wright PE, Dyson HJ (1999). Intrinsically unstructured proteins: re-assessing the protein structure-function paradigm. J Mol Biol..

[141] Dunker AK, Brown CJ, Lawson JD, Iakoucheva LM, Obradovic Z (2002). Intrinsic disorder and protein function. Biochemistry..

[142] Liu J, Tan H, Rost B (2002). Loopy proteins appear conserved in evolution. J Mol Biol..

[143] Iakoucheva LM, Brown CJ, Lawson JD, Obradović Z, Dunker AK (2002). Intrinsic disorder in cell-signaling and cancer-associated proteins. J Mol Biol..

[144] Coleman RG, Burr MA, Souvaine DL, Cheng AC (2005). An intuitive approach to measuring protein surface curvature. Proteins: Struct Funct Bioinform..

[145] Yuan Z, Zhao J, Wang Z-X (2003). Flexibility analysis of enzyme active sites by crystallographic temperature factors. Protein Eng..

[146] Baker NA, Sept D, Joseph S, Holst MJ, McCammon JA (2001). Electrostatics of nanosystems: application to microtubules and the ribosome. Proc Nat Acad Sci..

[147] Rocchia W, Alexov E, Honig B (2001). Extending the applicability of the nonlinear poisson-boltzmann equation: Multiple dielectric constants and multivalent ions. J Phys Chem B..

[148] Guerois R, Nielsen JE, Serrano L (2002). Predicting changes in the stability of proteins and protein complexes: a study of more than 1000 mutations. J Mol Biol..

[149] Schymkowitz J, Borg J, Stricher F, Nys R, Rousseau F, Serrano L (2005). The foldx web server: an online force field. Nucleic Acids Res..

[150] Cole C, Warwicker J (2002). Side-chain conformational entropy at protein–protein interfaces. Protein Sci..

[151] Yu C-M, Peng H-P, Chen C, Lee Y-C, Chen J-B, Tsai K-C (2012). Rationalization and design of the complementarity determining region sequences in an antibody-antigen recognition interface. PloS One..

[152] Laskowski RA, Thornton JM, Humblet C, Singh J (1996). X-site: use of empirically derived atomic packing preferences to identify favourable interaction regions in the binding sites of proteins. J Mol Biol..

[153] Zuckerkandl E, Pauling L (1965). Evolutionary divergence and convergence in proteins. Evolving Genes Proteins..

[154] Harms MJ, Thornton JW (2013). Evolutionary biochemistry: revealing the historical and physical causes of protein properties. Nat Rev Genet..

[155] de Juan D, Pazos F, Valencia A (2013). Emerging methods in protein co-evolution. Nat Rev Genet..

[156] Grishin NV, Phillips MA (1994). The subunit interfaces of oligomeric enzymes are conserved to a similar extent to the overall protein sequences. Protein Sci..

[157] Caffrey DR, Somaroo S, Hughes JD, Mintseris J, Huang ES (2004). Are protein–protein interfaces more conserved in sequence than the rest of the protein surface?. Protein Sci..

[158] Bradford JR, Westhead DR (2003). Asymmetric mutation rates at enzyme–inhibitor interfaces: implications for the protein–protein docking problem. Protein Sci..

[159] Reddy BV, Kaznessis YN (2005). A quantitative analysis of interfacial amino acid conservation in protein-protein hetero complexes. J Bioinform Comput Biol..

[160] Ma B, Elkayam T, Wolfson H, Nussinov R (2003). Protein–protein interactions: structurally conserved residues distinguish between binding sites and exposed protein surfaces. Proc Nat Acad Sci..

[161] Ofran Y, Rost B (2007). Protein–protein interaction hotspots carved into sequences. PLoS Comput Biol..

[162] Shoemaker BA, Zhang D, Tyagi M, Thangudu RR, Fong JH, Marchler-Bauer A (2012). Ibis (inferred biomolecular interaction server) reports, predicts and integrates multiple types of conserved interactions for proteins. Nucleic Acids Res..

[163] Wang B, Chen P, Huang D-S, Li J-J, Lok T-M, Lyu MR (2006). Predicting protein interaction sites from residue spatial sequence profile and evolution rate. FEBS Lett..

[164] Chelliah V, Chen L, Blundell TL, Lovell SC (2004). Distinguishing structural and functional restraints in evolution in order to identify interaction sites. J Mol Biol..

[165] Celniker G, Nimrod G, Ashkenazy H, Glaser F, Martz E, Mayrose I (2013). Consurf: using evolutionary data to raise testable hypotheses about protein function. Israel J Chem..

[166] Wilkins A, Erdin S, Lua R, Lichtarge O (2012). Evolutionary trace for prediction and redesign of protein functional sites. Comput Drug Discov Design..

[167] Glaser F, Pupko T, Paz I, Bell RE, Bechor-Shental D, Martz E (2003). Consurf: identification of functional regions in proteins by surface-mapping of phylogenetic information. Bioinformatics..

[168] Capra JA, Laskowski RA, Thornton JM, Singh M, Funkhouser TA (2009). Predicting protein ligand binding sites by combining evolutionary sequence conservation and 3d structure. PLoS Comput Biol..

[169] Berezin C, Glaser F, Rosenberg J, Paz I, Pupko T, Fariselli P (2004). Conseq: the identification of functionally and structurally important residues in protein sequences. Bioinformatics..

[170] Ponomarenko JV, Bourne PE (2007). Antibody-protein interactions: benchmark datasets and prediction tools evaluation. BMC Struct Biol..

[171] Dayhoff M, Schwartz R, Orcutt B (1978). A model of evolutionary change in proteins. Atlas Protein Seq Struct..

[172] Henikoff S, Henikoff JG (1992). Amino acid substitution matrices from protein blocks. Proc Nat Acad Sci..

[173] Shannon CE (1948). A mathematical theory of communication. Bell Syst Tech J..

[174] Mayrose I, Graur D, Ben-Tal N, Pupko T (2004). Comparison of site-specific rate-inference methods for protein sequences: empirical bayesian methods are superior. Mol Biol Evol..

[175] Glaser F, Rosenberg Y, Kessel A, Pupko T, Ben-Tal N (2005). The consurf-hssp database: the mapping of evolutionary conservation among homologs onto pdb structures. PROTEINS: Struct Funct Bioinform..

[176] Schneider R, Sander C (1996). The hssp database of protein structure-sequence alignments. Nucleic Acids Res..

[177] Kanamori E, Murakami Y, Tsuchiya Y, Standley DM, Nakamura H, Kinoshita K (2007). Docking of protein molecular surfaces with evolutionary trace analysis. Proteins: Struct Funct Bioinform..

[178] Lichtarge O, Bourne HR, Cohen FE (1996). An evolutionary trace method defines binding surfaces common to protein families. J Mol Biol..

[179] Lichtarge O, Sowa ME (2002). Evolutionary predictions of binding surfaces and interactions. Current Opin Struct Biol..

[180] Mihalek I, Reš I, Lichtarge O (2004). A family of evolution–entropy hybrid methods for ranking protein residues by importance. J Mol Biol..

[181] Chothia C, Lesk AM (1986). The relation between the divergence of sequence and structure in proteins. EMBO J..

[182] Lesk AM, Chothia C (1980). How different amino acid sequences determine similar protein structures: the structure and evolutionary dynamics of the globins. J Mol Biol..

[183] Petrey D, Fischer M, Honig B (2009). Structural relationships among proteins with different global topologies and their implications for function annotation strategies. Proc Nat Acad Sci..

[184] Ingles-Prieto A, Ibarra-Molero B, Delgado-Delgado A, Perez-Jimenez R, Fernandez JM, Gaucher EA (2013). Conservation of protein structure over four billion years. Structure..

[185] Kundrotas PJ, Zhu Z, Janin J, Vakser IA (2012). Templates are available to model nearly all complexes of structurally characterized proteins. Proc Nat Acad Sci..

[186] Monji H, Koizumi S, Ozaki T, Ohkawa T (2011). Interaction site prediction by structural similarity to neighboring clusters in protein-protein interaction networks. BMC Bioinformatics..

[187] Goncearenco A, Shoemaker BA, Zhang D, Sarychev A, Panchenko AR (2014). Coverage of protein domain families with structural protein-protein interactions: current progress and future trends. Progress Biophys Mol Biol..

[188] Khafizov K, Madrid-Aliste C, Almo SC, Fiser A (2014). Trends in structural coverage of the protein universe and the impact of the protein structure initiative. Proc Nat Acad Sci..

[189] Gao M, Skolnick J (2010). Structural space of protein–protein interfaces is degenerate, close to complete, and highly connected. Proc Nat Acad Sci..

[190] Tuncbag N, Gursoy A, Nussinov R, Keskin O (2011). Predicting protein-protein interactions on a proteome scale by matching evolutionary and structural similarities at interfaces using prism. Nat Protoc..

[191] Garma L, Mukherjee S, Mitra P, Zhang Y (2012). How many protein-protein interactions types exist in nature?. PloS One..

[192] Xie L, Xie L, Bourne PE (2011). Structure-based systems biology for analyzing off-target binding. Current Opin Struct Biol..

[193] Xie L, Bourne PE (2005). Functional coverage of the human genome by existing structures, structural genomics targets, and homology models. PLoS Comput Biol..

[194] Kundrotas PJ, Vakser IA (2010). Accuracy of protein-protein binding sites in high-throughput template-based modeling. PLoS Comput Biol..

[195] Koike A, Takagi T (2004). Prediction of protein–protein interaction sites using support vector machines. Protein Eng Des Sel..

[196] Glaser F, Steinberg DM, Vakser IA, Ben-Tal N (2001). Residue frequencies and pairing preferences at protein–protein interfaces. Proteins: Struct Funct Bioinform..

[197] Miller S (1989). The structure of interfaces between subunits of dimeric and tetrameric proteins. Protein Eng..

[198] Bouvier B, Grünberg R, Nilges M, Cazals F (2009). Shelling the voronoi interface of protein–protein complexes reveals patterns of residue conservation, dynamics, and composition. Proteins: Struct Funct Bioinformatics..

[199] Bordner AJ, Abagyan R (2005). Statistical analysis and prediction of protein–protein interfaces. Proteins: Struct Funct Bioinform..

[200] Prasad Bahadur R, Chakrabarti P, Rodier F, Janin J (2004). A dissection of specific and non-specific protein–protein interfaces. J Mol Biol..

[201] Bahadur RP, Chakrabarti P, Rodier F, Janin J (2003). Dissecting subunit interfaces in homodimeric proteins. Proteins: Struct Funct Bioinform..

[202] Hu Z, Ma B, Wolfson H, Nussinov R (2000). Conservation of polar residues as hot spots at protein interfaces. Proteins: Struct Funct Bioinform..

[203] Lukatsky D, Shakhnovich B, Mintseris J, Shakhnovich E (2007). Structural similarity enhances interaction propensity of proteins. J Mol Biol..

[204] Murakami Y, Jones S (2006). Sharp2: protein–protein interaction predictions using patch analysis. Bioinformatics..

[205] Negi SS, Schein CH, Oezguen N, Power TD, Braun W (2007). Interprosurf: a web server for predicting interacting sites on protein surfaces. Bioinformatics..

[206] Hamer R, Luo Q, Armitage JP, Reinert G, Deane CM (2010). i-patch: Interprotein contact prediction using local network information. Proteins: Struct Funct Bioinform..

[207] Warme PK, Morgan RS (1978). A survey of atomic interactions in 21 proteins. J Mol Biol..

[208] Xu D, Lin SL, Nussinov R (1997). Protein binding versus protein folding: the role of hydrophilic bridges in protein associations. J Mol Biol..

[209] Tsai C-J, Lin SL, Wolfson HJ, Nussinov R (1997). Studies of protein-protein interfaces: A statistical analysis of the hydrophobic effect. Protein Sci..

[210] Liu S, Zhang C, Zhou H, Zhou Y (2004). A physical reference state unifies the structure-derived potential of mean force for protein folding and binding. Proteins: Struct Funct Bioinform..

[211] Tsai C-J, Xu D, Nussinov R (1997). Structural motifs at protein-protein interfaces: Protein cores versus two-state and three-state model complexes. Protein Sci..

[212] McConkey BJ, Sobolev V, Edelman M (2003). Discrimination of native protein structures using atom–atom contact scoring. Proc Nat Acad Sci..

[213] Lawrence MC, Colman PM (1993). Shape complementarity at protein/protein interfaces. J Mol Biol..

[214] de Vries SJ, Bonvin AM (2008). How proteins get in touch: interface prediction in the study of biomolecular complexes. Current Protein Peptide Sci..

[215] Wass MN, David A, Sternberg MJ (2011). Challenges for the prediction of macromolecular interactions. Current Opinion Struct Biol..

[216] Yu L, Liu H (2004). Efficient feature selection via analysis of relevance and redundancy. J Mach Learn Res..

[217] Booker LB, Goldberg DE, Holland JH (1989). Classifier systems and genetic algorithms. Artif Intell..

[218] Andrew Moore MSL, Cohen WW, Hirsh H (1994). Efficient algorithms for minimizing cross validation error. Proceedings of the 11th International Confonference on Machine Learning.

[219] Matthews BW (1975). Comparison of the predicted and observed secondary structure of t4 phage lysozyme. Biochimica et Biophysica Acta (BBA)-Protein Struct..

[220] Maron O, Moore AW (1993). Hoeffding races: Accelerating model selection search for classification and function approximation. Adv Neural Inform Process Syst..

[221] Hoeffding W (1963). Probability inequalities for sums of bounded random variables. J Am Stat Assoc..

[222] Peng H, Long F, Ding C (2005). Feature selection based on mutual information criteria of max-dependency, max-relevance, and min-redundancy. Pattern Anal Mach Intell IEEE Trans..

[223] Ding C, Peng H (2005). Minimum redundancy feature selection from microarray gene expression data. J Bioinform Comput Biol..

[224] Cover TM, Thomas JA (1991). Entropy, relative entropy and mutual information. Elements Inform Theory.

[225] Li B-Q, Hu L-L, Niu S, Cai Y-D, Chou K-C (2012). Predict and analyze s-nitrosylation modification sites with the mrmr and ifs approaches. J Proteomics..

[226] Li B-Q, Hu L-L, Chen L, Feng K-Y, Cai Y-D, Chou K-C (2012). Prediction of protein domain with mrmr feature selection and analysis. PLoS One..

[227] Hotelling H (1933). Analysis of a complex of statistical variables into principal components. J Educ Psychol..

[228] Jolliffe I (2005). Principal component analysis. Encyclopedia of Statistics in Behavioral Science.

[229] Jackson DA. Stopping rules in principal components analysis: a comparison of heuristical and statistical approaches. Ecology. 1993:2204–14.

[230] Jones S, Thornton JM (1995). Protein-protein interactions: a review of protein dimer structures. Prog Biophys Mol Biol..

[231] De Vries SJ, Bonvin AM (2006). Intramolecular surface contacts contain information about protein–protein interface regions. Bioinformatics..

[232] Petrey D, Honig B (2002). Grasp2: visualization, surface properties, and electrostatics of macromolecular structures and sequences. Methods Enzymol..

[233] Yang A-S, Honig B (2000). An integrated approach to the analysis and modeling of protein sequences and structures. i. protein structural alignment and a quantitative measure for protein structural distance. J Mol Biol..

[234] Cortes C, Vapnik V (1995). Support-vector networks. Mach Learn..

[235] Jones DT, Taylor WR, Thornton JM (1992). The rapid generation of mutation data matrices from protein sequences. Comput Appl Biosci CABIOS..

[236] Gascuel O (1997). Bionj: an improved version of the nj algorithm based on a simple model of sequence data. Mol Biol Evol..

[237] Saitou N, Nei M (1987). The neighbor-joining method: a new method for reconstructing phylogenetic trees. Mol Biol Evol..

[238] Guindon S, Gascuel O (2003). A simple, fast, and accurate algorithm to estimate large phylogenies by maximum likelihood. Syst Biol..

[239] Breiman L (2001). Random forests. Mach Learn..

[240] Loriot S, Cazals F (2010). Modeling macro–molecular interfaces with intervor. Bioinformatics..

[241] Freund Y, Mason L (1999). The alternating decision tree learning algorithm. Proceedings of the Sixteenth International Conference on Machine Learning. ICML ’99.

[242] Pfahringer B, Holmes G, Kirkby R. Optimizing the induction of alternating decision trees. Adv Knowl Discov Data Mining. 2001:477–87.

[243] Aurenhammer F (1991). Voronoi diagrams—a survey of a fundamental geometric data structure. ACM Comput Surv (CSUR)..

[244] da Silveira CH, Pires DE, Minardi RC, Ribeiro C, Veloso CJ (2009). Protein cutoff scanning: A comparative analysis of cutoff dependent and cutoff free methods for prospecting contacts in proteins. Proteins: Struct Funct Bioinform..

[245] Fisher RA (1936). The use of multiple measurements in taxonomic problems. Ann Eugen..

[246] Venables WN, Ripley BD (2002). Modern applied statistics with s.

[247] Rumelhart DE, Hinton GE, Williams RJ (1986). Parallel distributed processing: Explorations in the microstructure of cognition, vol. 1.

[248] Haykin S, 1st edn. Upper Saddle River, NJ, USA: Prentice Hall PTR; 1994.

[249] Riedmiller M, Braun H (1993). A direct adaptive method for faster backpropagation learning: The rprop algorithm. Neural Networks, 1993., IEEE International Conference On.

[250] Breiman L (1996). Bagging predictors. Mach Learn..

[251] Fawcett T (2006). An introduction to roc analysis. Pattern Recognit Lett..

[252] Bradley AP (1997). The use of the area under the roc curve in the evaluation of machine learning algorithms. Pattern Recognit..

[253] Sørensen T (1948). A method of establishing groups of equal amplitude in plant sociology based on similarity of species and its application to analyses of the vegetation on danish commons. Biol Skr..

[254] Dice LR (1945). Measures of the amount of ecologic association between species. Ecology..

[255] Baldi P, Brunak S, Chauvin Y, Andersen CA, Nielsen H (2000). Assessing the accuracy of prediction algorithms for classification: an overview. Bioinformatics..

[256] Peng K, Obradovic Z, Vucetic S. Exploring bias in the protein data bank using contrast classifiers. In: Pacific Symposium on Biocomputing 2004: Hawaii, USA, 6-10 January 2004. World Scientific: 2003. p. 435. 10.1142/9789812704856_0041 14992523

[257] Kirchmair J, Markt P, Distinto S, Schuster D, Spitzer GM, Liedl KR, Langer T, Wolber G (2008). The protein data bank (pdb), its related services and software tools as key components for in silico guided drug discovery. J Med Chem..

[258] Bouman CA, Shapiro M (1994). A multiscale random field model for bayesian image segmentation. Image Process IEEE Trans..

[259] He X, Zemel RS, Carreira-Perpindn M (2004). Multiscale conditional random fields for image labeling. Computer Vision and Pattern Recognition, 2004. CVPR 2004. Proceedings of the 2004 IEEE Computer Society Conference On, vol. 2.

[260] Li X, Moal IH, Bates PA (2010). Detection and refinement of encounter complexes for protein–protein docking: taking account of macromolecular crowding. Proteins: Struct Funct Bioinform..

[261] Mnih V, Szepesvári C, Audibert J-Y (2008). Empirical bernstein stopping. Proceedings of the 25th International Conference on Machine Learning.

[262] Schölkopf B, Smola A, Müller K-R (1998). Nonlinear component analysis as a kernel eigenvalue problem. Neural Comput..

[263] Hinton GE, Salakhutdinov RR (2006). Reducing the dimensionality of data with neural networks. Science..

[264] Lise S, Archambeau C, Pontil M, Jones DT (2009). Prediction of hot spot residues at protein-protein interfaces by combining machine learning and energy-based methods. BMC Bioinformatics..

[265] Xu B, Wei X, Deng L, Guan J, Zhou S (2012). A semi-supervised boosting svm for predicting hot spots at protein-protein interfaces. BMC Syst Biol..

[266] Qi Y, Tastan O, Carbonell JG, Klein-Seetharaman J, Weston J (2010). Semi-supervised multi-task learning for predicting interactions between hiv-1 and human proteins. Bioinformatics..

[267] Bruzzone L, Persello C (2009). A novel context-sensitive semisupervised svm classifier robust to mislabeled training samples. Geosci Remote Sensing IEEE Trans..

[268] Frénay B, Verleysen M (2014). Classification in the presence of label noise: a survey. IEEE Trans Neural Netw Learn Syst..

[269] Tan A, Gilbert D, Deville Y (2003). Multi-class protein fold classification using a new ensemble machine learning approach. Genome Inform..

[270] Weston J, Watkins C. Support vector machines for multi-class pattern recognition, vol. 99. In: ESANN: 1999. p. 219–24.

[271] Moreira IS, Fernandes PA, Ramos MJ (2007). Hot spots—a review of the protein–protein interface determinant amino-acid residues. Proteins: Struct Funct Bioinform..

[272] Geppert T, Reisen F, Pillong M, Hähnke V, Tanrikulu Y, Koch CP (2012). Virtual screening for compounds that mimic protein–protein interface epitopes. J Comput Chem..

[273] Bogan AA, Thorn KS (1998). Anatomy of hot spots in protein interfaces. J Mol Biol..

[274] Livnah O, Stura EA, Johnson DL, Middleton SA, Mulcahy LS, Wrighton NC (1996). Functional mimicry of a protein hormone by a peptide agonist: the epo receptor complex at 2.8 å. Science..

[275] Johnson DL, Farrell FX, Barbone FP, McMahon FJ, Tullai J, Hoey K (1998). Identification of a 13 amino acid peptide mimetic of erythropoietin and description of amino acids critical for the mimetic activity of emp1. Biochemistry..

[276] Tinberg CE, Khare SD, Dou J, Doyle L, Nelson JW, Schena A (2013). Computational design of ligand-binding proteins with high affinity and selectivity. Nature..

[277] Schreiber G, Fleishman SJ (2013). Computational design of protein–protein interactions. Current Opin Struct Biol..

[278] De Vries SJ, van Dijk M, Bonvin AM (2010). The haddock web server for data-driven biomolecular docking. Nat Protocols..

[279] Dominguez C, Boelens R, Bonvin AM (2003). Haddock: a protein-protein docking approach based on biochemical or biophysical information. J Am Chem Soci..

[280] Lopes A, Sacquin-Mora S, Dimitrova V, Laine E, Ponty Y, Carbone A (2013). Protein-protein interactions in a crowded environment: an analysis via cross-docking simulations and evolutionary information. PLoS Comput Biol..

[281] Kuzu G, Keskin O, Gursoy A, Nussinov R (2012). Constructing structural networks of signaling pathways on the proteome scale. Current Opinion Struct Biol..

[282] Bandyopadhyay S, Sharan R, Ideker T (2006). Systematic identification of functional orthologs based on protein network comparison. Genome Research..

[283] Phan HT, Sternberg MJ (2012). Pinalog: a novel approach to align protein interaction networks—implications for complex detection and function prediction. Bioinformatics..

[284] Singh R, Xu J, Berger B (2008). Global alignment of multiple protein interaction networks with application to functional orthology detection. Proc Nat Acad Sci..

